# Loss of ATM accelerates pancreatic cancer formation and epithelial–mesenchymal transition

**DOI:** 10.1038/ncomms8677

**Published:** 2015-07-29

**Authors:** Ronan Russell, Lukas Perkhofer, Stefan Liebau, Qiong Lin, André Lechel, Fenja M Feld, Elisabeth Hessmann, Jochen Gaedcke, Melanie Güthle, Martin Zenke, Daniel Hartmann, Guido von Figura, Stephanie E Weissinger, Karl-Lenhard Rudolph, Peter Möller, Jochen K Lennerz, Thomas Seufferlein, Martin Wagner, Alexander Kleger

**Affiliations:** 1 Department of Internal Medicine I, Ulm University, Albert-Einstein-Allee 23, Ulm 89081, Germany; 2 Institute of Neuroanatomy, Eberhard Karls University Tuebingen, Oesterbergstr. 3, Tuebingen 72074, Germany; 3 Department of Cell Biology, Institute for Biomedical Engineering, Medical Faculty, RWTH Aachen University, Pauwelstr. 30, Aachen 52074, Germany; 4 Institute of Pathology, Ulm University, Albert-Einstein-Allee 23, Ulm 89081, Germany; 5 Department of Gastroenterology II, University Medical Center Goettingen, Robert-Koch-Str. 40, Goettingen 37075, Germany; 6 Department of Surgery, University Medical Center Goettingen, Robert-Koch-Str. 40, Goettingen 37075, Germany; 7 Department of Surgery, Technische Universität München, Ismaninger Str. 22, Munich 81675, Germany; 8 II. Medizinische Klinik, Klinikum rechts der Isar, Technische Universität München, Ismaninger Str. 22, Munich 81675, Germany; 9 Leibniz Institute for Age Research - Fritz Lipmann Institute e.V., Beutenbergstr. 11, Jena 07745, Germany

## Abstract

Pancreatic ductal adenocarcinoma (PDAC) is associated with accumulation of particular oncogenic mutations and recent genetic sequencing studies have identified ataxia telangiectasia-mutated (ATM) mutations in PDAC cohorts. Here we report that conditional deletion of ATM in a mouse model of PDAC induces a greater number of proliferative precursor lesions coupled with a pronounced fibrotic reaction. ATM-targeted mice display altered TGFβ-superfamily signalling and enhanced epithelial-to-mesenchymal transition (EMT) coupled with shortened survival. Notably, our mouse model recapitulates many features of more aggressive human PDAC subtypes. Particularly, we report that low expression of ATM predicts EMT, a gene signature specific for Bmp4 signalling and poor prognosis in human PDAC. Our data suggest an intimate link between ATM expression and pancreatic cancer progression in mice and men.

## Abstract (web summary)

Mutations in the serine/threonine kinase ataxia teleangiectasia mutated (ATM) have been linked to pancreatic ductal adenocarcinoma (PDAC) cohorts. Here Russell *et al*. show that loss of ATM induces a greater number of proliferative precursor lesions in a mouse model, recapitulating many features of human PDAC subtypes.

## Graphical abstract

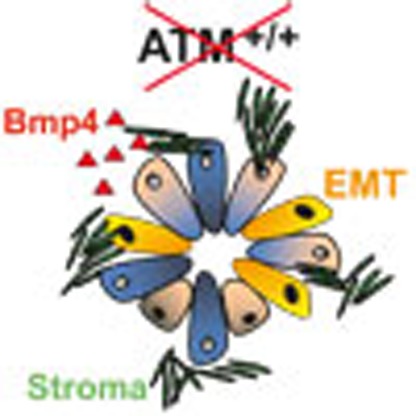

Despite intensive basic and clinical research, deaths due to pancreatic ductal adenocarcinoma (PDAC) rank fourth among cancer-related events in the western world, with an overall 5-year survival rate around 4% (ref. 1). Genetically engineered animal models effectively recapitulate both the morphological and molecular features of PDAC and have helped to identify key factors within the genetic landscape directing PDAC formation such as oncogenic *K-ras*, *Trp53* and *Ink4A/Arf.* This has led to a better understanding of the molecular mechanisms that drive PDAC. Genome-wide exome-sequencing studies recently identified several novel mutations associated with PDAC^2^,^3^,^4^,^5^. However, the precise contribution of these newly identified factors within tumour biology remains elusive.

Ataxia telangiectasia mutated (ATM) is a serine/threonine kinase and was initially characterized for its role in the DNA damage response (DDR)^6^,^7^,^8^. Interestingly, accumulating evidence suggests that ATM has a broader capacity to integrate and direct various signalling cues to maintain cellular homeostasis than previously appreciated^9^,^10^. These include regulation of chromatin remodelling, oxidative stress, and cellular metabolism in diverse tissues. Patients with the recessive disease ataxia telangiectasia (AT) and ATM-deficient mice exhibit immunodeficiency, genomic instability, and an increased risk for lymphoid malignancies. Moreover, ATM deficiency has an impact on self-renewal of hematopoietic stem cells, accelerates ageing in telomere dysfunctional mice, and drives angiogenesis^9^. Thus, it is becoming increasingly clear that ATM serves multiple functions in a variety of cellular compartments. To date, the role of ATM in pancreatic cancer initiation/progression is largely unclear. Inactivating variants of the ATM gene are carried by ∼1% of the general population^11^ and more recently, familial pancreatic cancer patients have been shown to harbour a nonsense ATM germ line mutation leading to somatic loss of the variant allele^12^,^13^. Recent data based on large-scale sequencing studies reported up to 18% of ATM mutations in certain human PDAC cohorts, which can be even detected in the germ line of certain individuals^3^,^5^,^14^,^15^,^16^. In line, a recent report underscored this finding on the protein level^17^. This observation positions ATM next to established factors such as *K-ras* or *Tr53* among the 16 most commonly mutated genes in PDAC^5^,^15^,^16^.

Herein we examine the role of ATM in PDAC tumour biology in both mice and men and provide evidence that loss of ATM (1) enhances acinar-to-ductal reprogramming (ADR) via altered TGFβ-superfamily signalling, (2) is associated with epithelial-to-mesenchymal transition (EMT) and a gain in tumour initiating properties and (3) acts as an independent prognostic marker as ATM depletion correlates significantly with survival.

## Results

### Loss of ATM promotes ADR and neoplastic lesion formation

First, we reanalysed currently available information on ATM mutations from two comprehensive PDAC data sets from the International Cancer Genome Consortium. These independent cohorts from Canada and Australia show mutation frequencies of 9-18% averaging to about 12% within the ATM gene. This includes non-synonymous substitutions and insertion-deletions (indels) and in line with recent reports, underscores the clinical and biological relevance of *ATM* mutations in a significant subset of human pancreatic cancers (Table 1)^3^,^5^,^14^,^16^,^17^.

**Figure 1:**
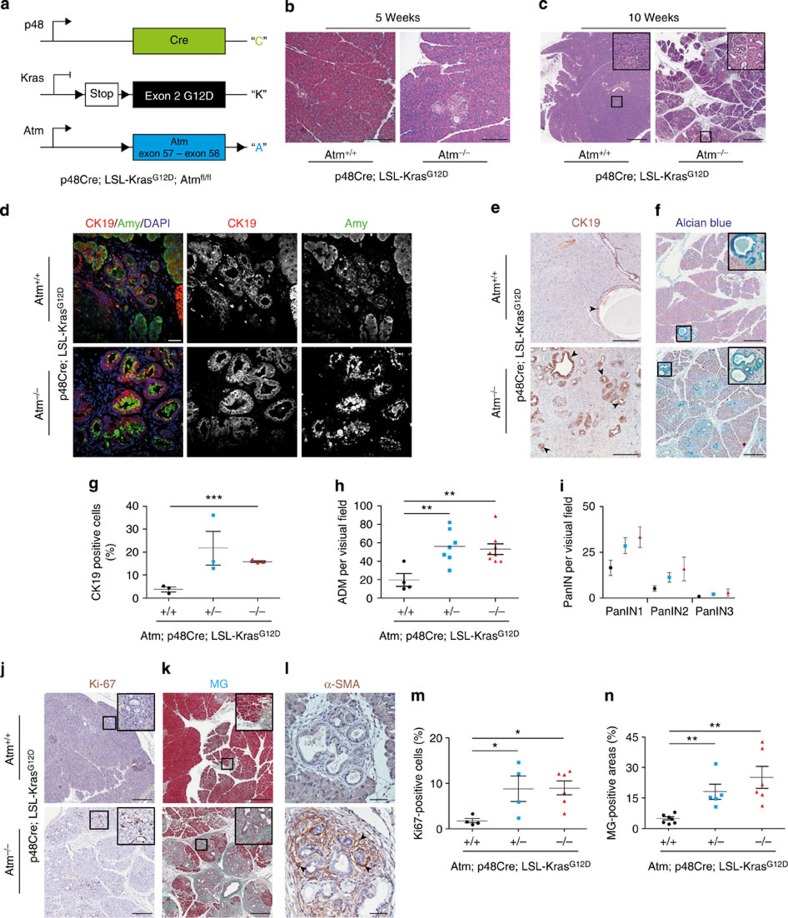
ATM loss of function promotes neoplastic changes in the pancreas in the context of oncogenic K-ras. (**a**) Illustration of strategy to generate p48Cre;Kras^G12D/+^;Atm^−/−^ mice (p48^Cre/+^=“C”; Kras ^G12D/+^=“K”; Atm^−/−^=“A”). (**b**,**c**) Representative haematoxylin and eosin (H&E)-stained sections of pancreas at the indicated time points (**b**) scale bar, 200 μm, (**c**) scale bar, 500 μm. (**d**) Immunofluorescence staining of pancreas from the respective genotypes at 10 weeks old shows expression of CK19 (red), amylase (green) and nuclei (Dapi-blue). Scale bar, 20 μm. (**e**,**f**) Immunohistochemistry shows expression of (**e**) CK19 (scale bar, 200 μm) and (**f**) alcian blue (scale bar, 500 μm) in precursor lesions. Scale bar, 20 μm, (**g**–**i**) Quantification of (**g**) CK19-positive cells, (**h**) ADM events per visual field, (**i**) PanIN grading and numbers are shown according to the genotype at 10 weeks. Colour code: Black=Atm^+/+^; Blue =Atm^+/−^ and Red=Atm^−/−^. (**j**–**l**) Immunohistochemical staining reveals (**j**) Ki67 (scale bar, 500 μm), (**k**) fibrosis (Masson–Goldner) (scale bar, 500 μm) and (**l**) α-SMA (scale bar, 20 μm) at sites of pre-malignant lesions in pancreata from the indicated genotypes and respective quantifications: (**m**,**n**). Representative images from at least three mice per genotype are shown. *P<0.05, **P<0.01, ***P<0.0001. One-way analysis of variance (ANOVA). Error bars are the means±s.e.m.

To examine the role of ATM deficiency in pancreatic carcinogenesis, we crossed mice harbouring a floxed *Atm* (‘A') allele to an existing PDAC model, p48^Cre/+^ (‘C'), Kras^G12D/+^ (‘K') mice (Fig. 1a)^18^. Animals containing all three alleles are referred to as AKC mice throughout the text and characterization of target animals is described in detail in Supplementary Fig. 1a–d. Of note, animals with loss of ATM alone showed normal pancreatic development at all time points examined (Supplementary Fig. 1e). Initial analysis on mice at 5 weeks of age revealed a small number of foci with altered acinar architecture within the pancreas of KC mice. In contrast, age-matched AKC animals already showed more parenchymal foci with disruption of acinar tissue (Fig. 1b).

The early stages of human PDAC are characterized by the onset of defined ductal precursor lesions, the so-called acinar-to-ductal metaplasias (ADMs), acinar-to-ductal reprogramming (ADRs) and pancreatic intraepithelial neoplasias (PanINs)^18^,^19^. At 10 weeks of age, we observed more progressive loss of Amylase+ acinar tissue accompanied by an increase in Cytokeratin 19 (CK 19)+ or alcian blue+ ductal precursor lesions in AKC mice compared with KC controls (Fig. 1c,d). Carboxypeptidase (CPA) and SOX9 immunostaining confirmed this finding (Supplementary Fig. 2a). Specifically, we observed a predominant increase of ADM lesions (Fig. 1d,h and Supplementary Fig. 2a) and low-grade PanINs but also increased numbers of high-grade PanINs in AKC mice compared with KC mice (Fig. 1e–g,i). Of note, the most striking changes in pancreatic architecture occurred in the head and upper-body region of the pancreas, while the lower body and tail was generally undisturbed. As previously described for ATM, the phenotype of heterozygous- and homozygous-depleted ATM animals was similar, indicating that haploinsufficiency is sufficient to cause a pancreatic phenotype^20^,^21^,^22^,^23^.

ATM is a well-known cell cycle checkpoint^10^. Therefore, we checked whether there is any change in the expression of markers and known regulators of the cell cycle. Immunohistochemistry (IHC) for Ki-67, CyclinD1 and CyclinE revealed a higher number of proliferating cells within the ductal precursor lesions of ATM-targeted mice (Fig. 1j,m and Supplementary Fig. 2b,c) compared with controls. Furthermore, Masson–Goldner staining revealed a significant amount of extracellular matrix production particularly in areas of ADM lesions. Intriguingly, ADM lesions of AKC mice showed more α-SMA staining within the areas of neoplastic lesions (Fig. 1k,l,n), indicative of activated pancreatic stellate cells as the major source of stromal infiltration.

### Loss of ATM enhances EMT and stemness

ATM is a central mediator of the DNA-damage response (DDR), which acts as a barrier against tumour progression^24^. Interestingly, downstream targets of ATM (such as p21 and γH2AX) as well as resulting senescence were not appreciably altered in AKC mice compared with KC mice, while levels of p53 were increased within the precursor lesions of AKC mice (Supplementary Fig. 2d–f). Levels of apoptosis and numbers of inflammatory cells also did not differ (Supplementary Fig. 2g,h).

**Figure 2:**
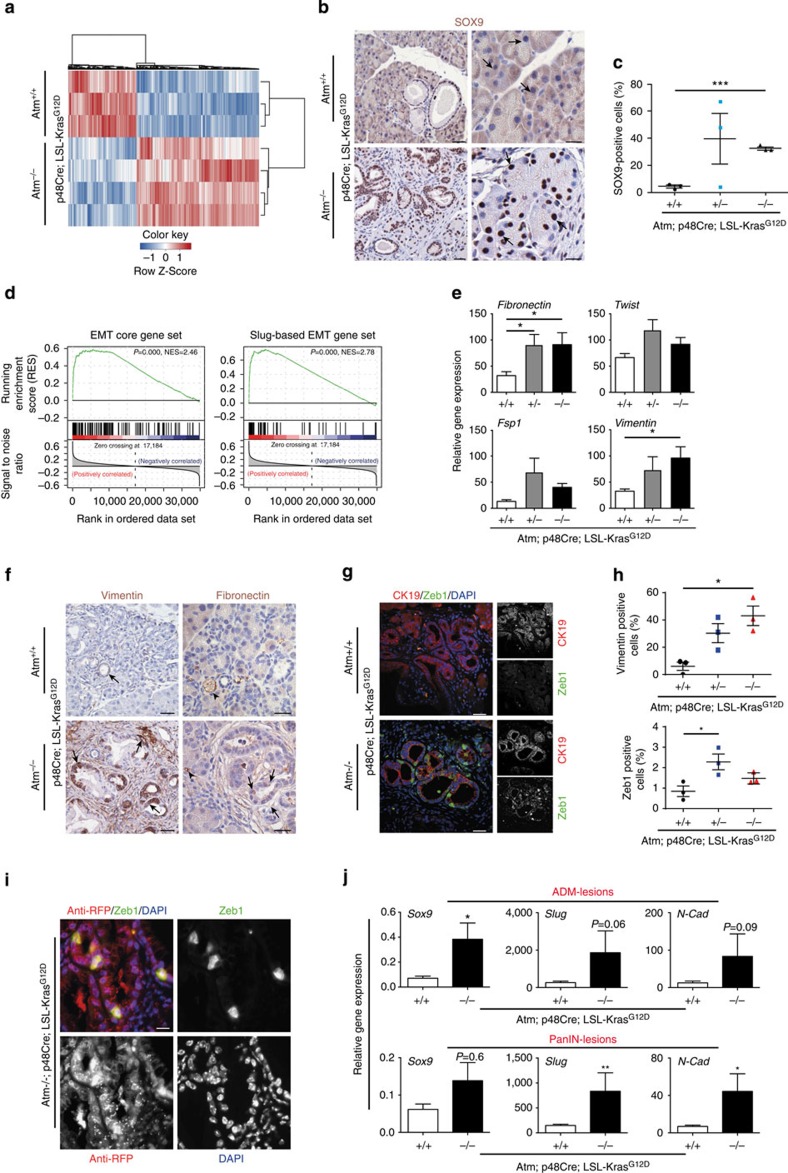
Epithelial-to-mesenchymal transition (EMT) is accelerated in ATM-targeted pancreata. (**a**) Genome-wide transcriptional profiling identified 2,472 differentially regulated genes shown as a hierarchically clustered heat map of pancreata from 10 week aged p48^Cre/+^;Kras^G12D/+^;Atm^−/−^(AKC) - and p48^Cre/+^;Kras^G12D/+^;Atm^+/+^(KC)- mice. (**b**,**c**) Immunohistochemistry (left panel, scale bar, 20 μm, right panel, scale bar, 10 μm) and quantitative analysis (**c**) of Sox9 positivity in precursor lesions. Representative images of normal acini of the respective genotypes are also shown to illustrate ductal programming in AKC mice (**B**, right panel). (**d**) GSEA of differentially regulated genes from (**a**) shows enrichment of EMT-associated genes in p48^Cre/+^;Kras^G12D/+^;Atm^−/−^ pancreata using two independent ‘GSE sets'^28^,^29^. (**e**) RT-qPCR showing increased levels of *Fibronectin*, *Twist1*, *Fsp1* and *Vimentin* in the pancreas of p48^Cre/+^;Kras^G12D/+^;Atm^−/−^ and p48^Cre/+^;Kras^G12D/+^;Atm^+/−^ versus controls (*n*=5 per group). Student's *t*-test *P<0.05. Error bars, s.e.m. (**f**) Immunohistochemistry staining shows expression of Vimentin and Fibronectin in precursor lesions. Scale bar, 20 μm. (**g**) Immunofluorescence stainings reveal more abundant expression of Zeb1-positive cells in CK19-positive precursor lesions of AKC-pancreata compared with controls. Scale bar, 20 μm. (**h**) Quantitative analysis of vimentin and Zeb1-positive cells in precursor lesions in the, respective, genotypes. (**i**) Immunofluorescence staining in p48^Cre/+^;Kras^G12D/+^;Atm^−/−^;Rosa_tdRFP^fl/fl^ mice against RFP (red) and ZEB1 (green) and Dapi (blue). Scale bar, 10 μm. (**j**) RT-qPCR analysis showing expression levels of *Sox9* (ADM: *n*=4 versus 6; PanIN: *n*=4 versus 5), *Slug* (ADM: *n*=4 versus 6; PanIN: *n*=4 versus 6) and *N-cadherin* (ADM: *n*=3 versus 6; PanIN: *n*=3 versus 6) in microdissected ADM or PanIN lesions from p48^Cre/+^;Kras^G12D/+^;Atm^+/+^ and p48^Cre/+^;Kras^G12D/+^;Atm^−/−^ animals, respectively. Mann–Whitney test was used for statistical analysis. *P<0.05, **P<0.01. Error bars, s.e.m.

To gain further insights into the molecular changes of ATM depletion, we performed comparative genome-wide transcriptional profiling followed by gene set enrichment analysis. Using age-matched (∼10 weeks) KC and AKC mice a set of 2,472 differentially regulated genes was identified between the two groups, many of which have previously been implicated in PDAC (Fig. 2a). In addition, we identified a strong deregulation among collagen and matrix-metalloproteinase (MMP) family members (Supplementary Fig. 3a,b) in line with our observation of a pronounced desmoplastic reaction. SOX9 acts as the driving force of acinar-to-ductal metaplasia (ADM) and acinar-to-ductal reprogramming (ADR) in the pancreas^19^. Consistent with this, we found a pronounced upregulation of Sox9 in ATM-depleted mice, in morphologically intact acinar tissue and also in areas of ADM/ADR pointing towards enhanced ductal programming of acinar structures (Fig. 2b,c and Supplementary Fig. 2a).

Certain types of cancers undergo epithelial-to-mesenchymal transition (EMT), a process highly abundant during embryonic development. Interestingly, EMT is particularly prevalent in areas of ADM^25^ and many factors associated with desmoplasia induce EMT-associated transcription factors (EMT-TFs) such as *SOX9* or *SLUG*^26^,^27^. To assess on-going EMT in our model, gene set enrichment analysis (GSEA) of differentially regulated genes between AKC and KC mice was plotted against published gene sets that are implicated in the EMT process^28^,^29^. We found that AKC mice present a robust gene signature associated with EMT, as identified using two independent ‘GSEA-core' sets (Fig. 2d, Supplementary Fig. 3f)^28^,^29^. Of note, 10 random gene sets each containing 100 genes randomly selected from the mouse genome were subjected to GSEA and all the *P* values are above 0.05, suggesting no similarity or bias to any group of samples (Supplementary Fig. 3d,e). Next, we corroborated the GSEA data via qPCR analysis for *Fibronectin*, *Twist1*, *FSP1* and *Vimentin* (Fig. 2e). Further evidence for increased EMT in precursor lesions of AKC mice is given by IHC for Vimentin and Fibronectin and immunofluorescence staining for ZEB1 (Fig. 2f–h). Taken together, this implies that a significant proportion of ductal epithelial cells in the pancreas of AKC animals have acquired mesenchymal features compared with age-matched KC mice.

To further substantiate these findings and to demonstrate that the observed increase in mesenchymal features is not solely due to increased desmoplasia within the AKC mice, we crossed the AKC mice with Rosa26-tdRFP mice^30^. This allowed us to trace the cells in which recombination took place and essentially provide a system whereby the epithelial cells of the pancreas could be identified clearly from the surrounding stroma. IHC analysis for the RFP in combination with Zeb1 provides qualitative evidence that indeed the neoplastic lesions, which originate from the epithelium of the pancreas, undergo EMT (Fig. 2i, Supplementary Fig. 4a,b).

To provide a quantitative analysis for EMT between KC and AKC mice, we adopted a strategy that allowed us to isolate a purified population of ductal and neoplastic ductal cells using a DBA-lectin sorting as previously reported^31^. We first validated this approach as shown in Supplementary Fig. 4d, whereby ductal components, which express Ck19, are enriched in the DBA+ population. Subsequently, by depleting the contaminating stroma and other cell types and enriching for the ductal components, we could confirm that indeed neoplastic duct-like cells of the AKC mice express more markers of EMT (Supplementary Fig. 4c,e). Finally, using micro-dissection techniques to specifically isolate the different neoplastic compartments within the pancreata at 18 weeks of age, we validated our previous observations that AKC mice exhibit more EMT than KC mice. As shown in Fig. 2j, both ADM and PanIN lesions express higher mRNA levels of the EMT-associated genes *Sox9*, *Slug* and *N-cadherin*.

**Figure 3:**
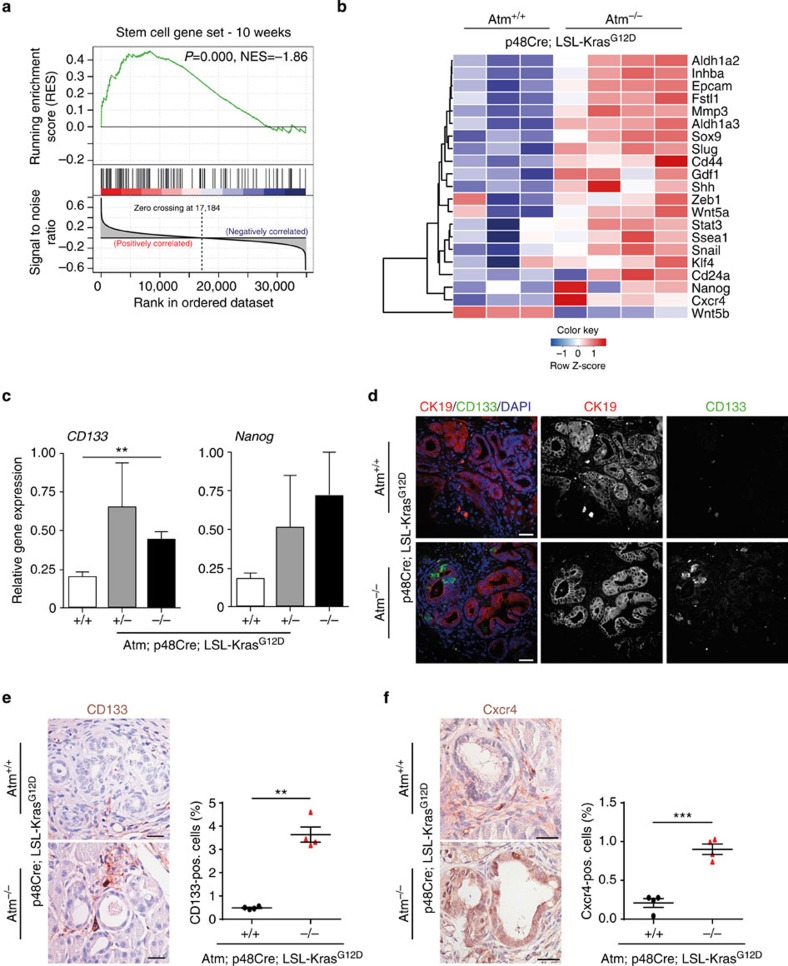
ATM depletion enriches for cancer stem cells and associated signalling pathways. (**a**) Gene set enrichment analysis of differentially regulated genes from (Fig. 2a) identifies enrichment of a stem cell associated gene set^33^ in AKC pancreata at 10 weeks of age. (**b**) Hierarchically clustered heat map illustration shows differential expression of stemness-associated genes among AKC mice. (**c**) RT-qPCR showing increased levels of *CD133* and *Nanog* in the pancreas of AKC mice versus controls. (**d**) Immunofluorescence staining of pancreata from the respective genotypes at 10 weeks old shows expression of CK19 (red), CD133 (green) and nuclei (Dapi-blue). Scale bar, 20 μm. (**e**,**f**) IHC staining and quantifications for CD133 (scale bar, 20 μm) (**e**) and Cxcr4 (scale bar, 10 μm) (**f**) in the respective genotypes.

Recently, it has been demonstrated that the acquisition of EMT is associated with cancer stem cells (CSCs), which contribute to the progression of human cancers^32^. Similar to embryonic stem cells, CSCs exhibit self-renewal and extended differentiation capacity but are also thought to represent the major source of migratory cells with invasive potential. Interestingly, the presence of a CSC gene expression signature in colorectal cancer strongly predicts poor patient's survival^33^,^34^. In line with on-going EMT, GSEA revealed an over-representation of stem cell-associated genes in AKC mice (Fig. 3a) such as *Epcam*, *Sox9*, *Slug*, *Snail*, *Stat3*, *Klf4* or the recently identified Mmp3-Wnt5b axis (Fig. 3b). To further substantiate this finding, qPCR analysis showed higher *CD133* and *Nanog* levels (Fig. 3c), and immunofluorescence staining revealed higher numbers of CD133-positive cells^35^ at the border of ADMs in AKC mice at 10 weeks of age (Fig. 3d,e). In line, Cxcr4, a marker labelling invasive cancer stem cells^35^, is also increased in AKC mice (Fig. 3f). Further evidence is provided by a hyperactive NODAL/SMAD2 signalling axis in AKC mice identified by both GSEA^36^ and immunoblotting for Nodal and phosphorylated SMAD2/3 (Supplementary Fig. 4f,g,h). Of note, a recent study shows that the overexpression of Nodal induces a metastatic phenotype in pancreatic cancer cells via the Smad2/3 pathway^37^.

### Acinar cell de-differentiation via enhanced BMP4 signalling

**Figure 4:**
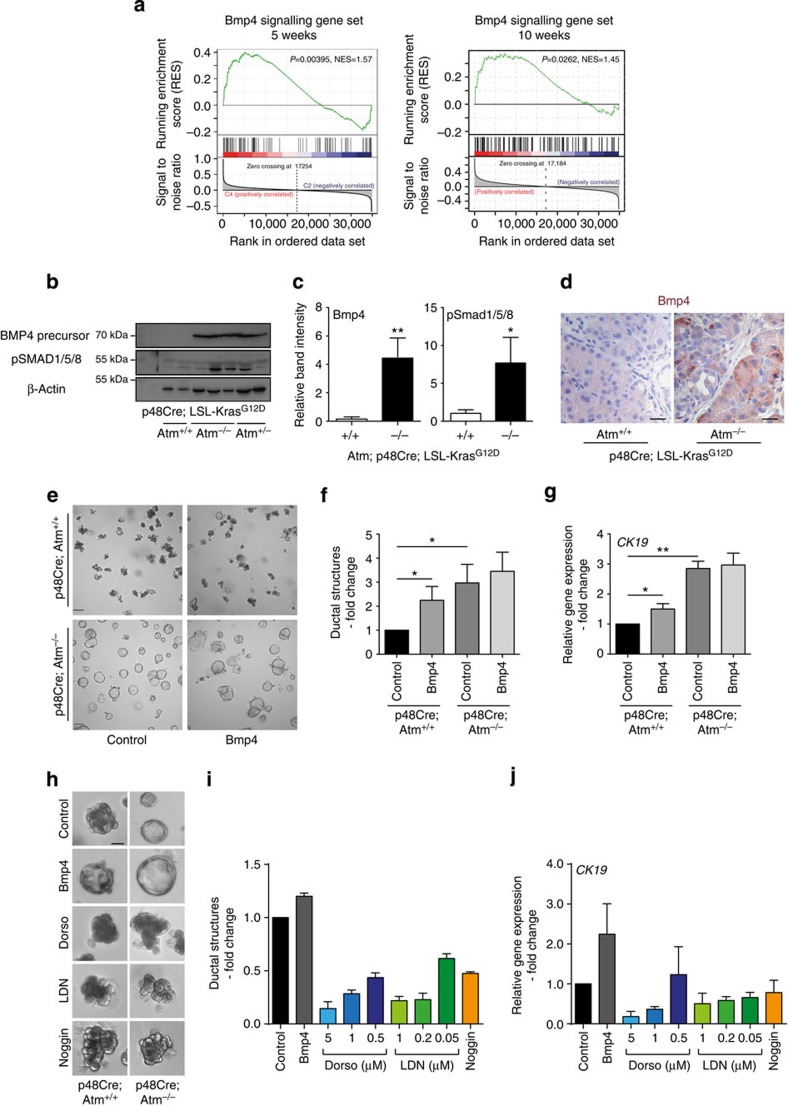
Loss of ATM activity compromises acinar cell integrity. (**a**) GSEA of the differentially regulated genes from the respective genotypes identifies enrichment of the BMP4 signalling signature^42^ in p48^Cre/+^;Kras^G12D/+^;Atm^−/−^ pancreata at 5 weeks and 10 weeks of age. (**b**) Immunoblot of BMP4, Phospho-Smad 1/5/8 and β-actin in the respective genotypes. (**c**) Quantification of several immunoblots as representative images in **b** for the following mouse numbers. Bmp4: 4 KC versus 6 AKC animals. SPmad 1/5/8: 4 KC versus 5 AKC animals. Mann–Whitney test was used for statistical analysis. *P<0.05, **P<0.01. Error bars, s.e.m. (**d**) Bmp4 staining of KC and AKC mice pancreata shows predominant Bmp4 staining in the acinar compartment of AKC mice. Staining is representative for at least three mice per group. Scale bar, 10 μm. (**e**) Bright-field images of acinar cell cultures from freshly isolated acini cultured for 2 days in growth factor reduced matrigel under indicated conditions. BMP4 was used at 25 ng ml^−1^; *n*=4. Scale bar, 50 μm. (**f**,**g**) Quantification of ductal structures at day 2 of culture and RT-qPCR showing levels of the ductal gene marker *CK19* in the respective conditions. Fold changes were calculated by setting levels in control treated Atm^+/+^ acini to 1. Mann–Whitney test was used for statistical analysis. *P<0.05, **P<0.01. Error bars, s.e.m. (**h**) High-power bright-field images of acinar cell cultures under the indicated conditions at day 2 of culture. Scale bar, 10 μm. (**i**) Quantification of ductal structures at day 2 and (**j**) RT-qPCR analysis showing levels of the ductal marker gene *CK19* in the respective conditions from Atm^−/−^ acini (2 out of 3 experiments with similar results are shown). Error bars, s.e.m. All analyses were performed on 4- to 6-week-old animals.

EMT is associated with secretion and accumulation of growth factors that have a profound influence on the tumour microenvironment and support tumour growth^26^. Numerous signalling pathways, including BMPs and Nodal^38^,^39^,^40^, are known to converge in early development to direct EMT and cell lineage commitment^41^. GSEA of differentially regulated genes between AKC and KC mice identified a hyperactive BMP4 pathway in AKC mice (Fig. 4a, Supplementary Fig. 3c)^42^. Interestingly, we identified an enrichment of both Bmp4 signalling and EMT factors and EMT-signalling gene sets in AKC mice at the 5 weeks stage, underpinning the contribution of these factors to disease initiation and progression (Fig. 4a, Supplementary Fig. 3f). This was further substantiated by western blot analysis with significantly increased BMP4 precursor protein and phosphorylated SMAD1/5/8-levels observed in the pancreas of AKC mice (Fig. 4b,c). IHC analysis spatially localized the increased BMP4 signal in AKC mice to the acinar compartment, thereby pointing towards the acini as the primary source for local BMP4 activity in AKC mice (Fig. 4d). Recently, the BMP4/SMAD1/MMP2 axis has been identified to mediate EMT and subsequent invasive properties in PDAC^40^. Indeed, MMP2 is upregulated in AKC mice as confirmed via microarray and immunoblotting from whole pancreatic lysate (Supplementary Fig 3a,g).

To directly assess the propensity of ATM-deficient acini to trans-differentiate to duct-like cells, acinar cell clusters were explanted and grown in matrigel layers *in vitro*. Of note, these experiments were performed in ATM-targeted mice and control mice in the absence of oncogenic Kras to more accurately define the role of ATM loss as a single event. Isolation of the acinar cell compartment was achieved with ∼90% cell viability (data not shown) from both control and AC mice. At day 1 of culture, no significant differences in acinar cell morphology were observed (Supplementary Fig. 5a). Remarkably, however, acini from AC mice underwent significantly more ductal reprogramming compared with controls by day 2 (Fig. 4e–g). Thus, ATM loss as a single event facilitates ductal programming. On the basis of our observation that BMP signalling was deregulated in AKC mice, we included BMP4 and inhibitors of the BMP4 signalling axis—dorsomorphin and LDN 193189^43^—to assess their effects on the *in vitro* trans-differentiation capacity of acinar explants. Notably, addition of BMP4 to the acinar explant cultures significantly increased the trans-differentiation of acinar cells from control mice. In line with the observation that the acinar compartment of AKC mice is endogenously saturated with Bmp4, we only observed a mild increase of ductal structures in ATM-depleted acini (Fig. 4f,g). Notably, BMP4 treatment gave rise to larger and more irregular ductal structures than in control conditions (Fig. 4e,h). In contrast chemical inhibition of endogenous BMP4 signalling via dorsomorphin or LDN-193189 prevented ductal programming in AC mice-derived acini in a dose-dependent manner, demonstrating the central role of this pathway in the acinar trans-differentiation process (Fig. 4h–j, Supplementary Fig. 5b,c). These observations were confirmed via qPCR analysis, which demonstrated that inhibitors prevent *CK19* expression while it stabilizes the acinar cell phenotype as shown by high expression of *Amylase* (Fig. 4i,j and Supplementary Fig. 5b). Similar results were also obtained using recombinant human Noggin^44^ to inhibit mostly BMP4 activity (Fig. 4h–j, Supplementary Fig. 5c). Taken together, this *ex vivo* culture system reiterates the effect of *ATM* gene disruption on acinar cell integrity as seen *in vivo* and highlights the importance of the BMP4 signalling axis within this process.

### Loss of ATM limits survival in PDAC in mice and men

**Figure 5:**
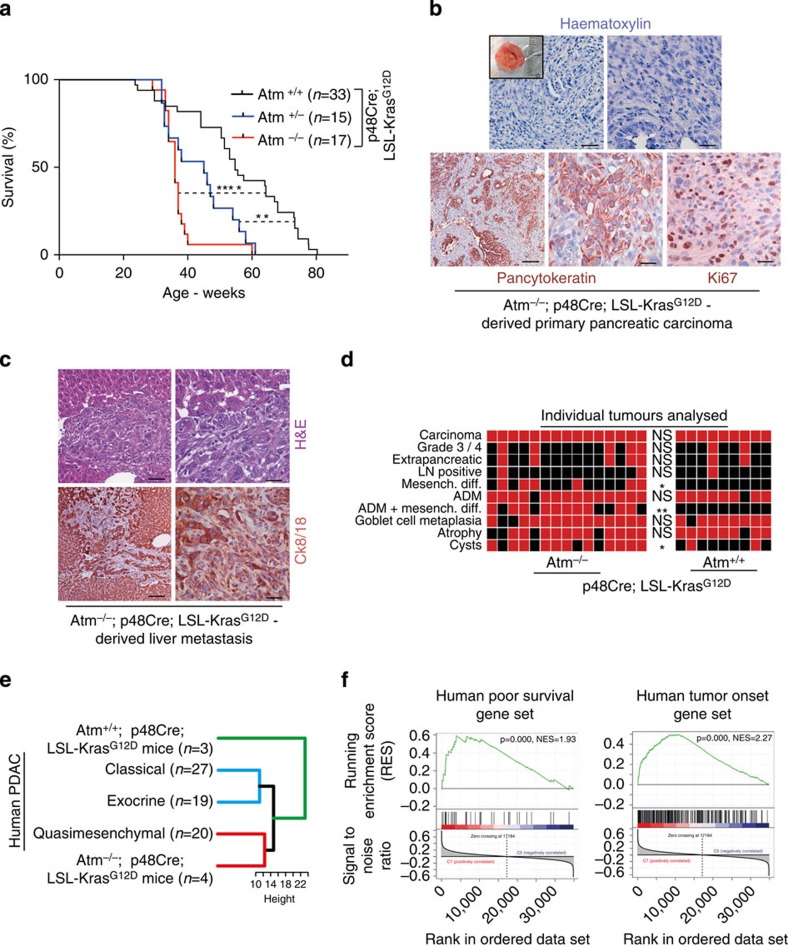
ATM deficiency reduces PDAC survival in mice and men. (**a**) Kaplan–Meier analysis of survival of p48^Cre/+^;Kras^G12D/+^;Atm^−/−^ and p48^Cre/+^;Kras^G12D/+^;Atm^+/-^ and p48Cre;Kras^G12D/+^;Atm^+/+^ mice. Ageing target mice were killed upon obvious signs of wasting. Log-rank (Mantel-Cox) test **P<0.01, ****P<0.0001. (**b**) Representative images of tumours arising in p48^Cre/+^;Kras^G12D/+^;Atm^−/−^ mice (top panel: inlet: macroscopic tumour, left image: low-power (scale bar, 50 μm), right image: high-power (scale bar, 20 μm) (lower panel: Ck19 stained tumour, left image: low-power (scale bar, 200 μm), middle image: high power (scale bar, 20 μm) and right image: IHC for ki67 stained tumour (scale bar, 20 μm)). (**c**) An example of an associated liver metastasis stained for H&E and Ck8/18. Left panel, scale bar, 50 μm; right panel, scale bar, 20 μm. (**d**) Heat map depicting tumour characteristics from individual mice of the respective genotypes. Black=negative; Red=positive. (**e**) Hierarchical clustering using Euclidian distance shows that pancreata from p48^Cre/+^;Kras^G12D/+^;Atm^−/−^ mice cluster more closely with human PDACs having a quasi-mesenchymal subtype. p48^Cre/+^;Kras^G12D/+^;Atm^+/+^ mice pancreata cluster as described previously more with the ‘classical' subtype PDAC^48^. (**f**) GSEA plots show an abundance of tumour onset and poor survival gene sets in AKC pancreata^50^,^51^.

Kaplan–Meier survival analysis revealed a significantly reduced survival of ATM-deficient mice (AKC), with a median survival of 36 and 45 weeks for homozygous and heterozygous ATM-targeted animals respectively, compared with 55 weeks for KC mice (Fig. 5a). Remarkably, both homozygous and heterozygous AKC mice developed highly proliferative tumours at an earlier time point than control animals. Microscopically invasive tumour fractions were rich in spindle-shaped tumour cells pointing to a mesenchymal pattern. In addition, we found large amounts of adjacent ADM lesions^45^ and the number of dysplastic cysts^46^,^47^ was higher in AKC mice (Fig. 5b,d). Liver metastasis was found in 6 out of 32 AKC mice (Fig. 5b,c), while this occured only once in 33 KC mice.

Recently, human PDAC was classified by a genetic profile into three distinct subtypes: classical (epithelial-type), quasi-mesenchymal (QM) and exocrine-like. Of note, patients with QM-PDAC were found to have the worst prognosis and a dedifferentiated phenotype^48^. We compared the gene expression profile of 10-week-old AKC and KC mice with these human PDAC signatures^48^,^49^. Hierarchical clustering using Euclidean distance revealed closer association of AKC mice pancreata with the QM human PDACs compared with KC mice (Fig. 5e). We also found an independent human gene expression signature of 36 genes indicating poor prognosis in PDAC patients strongly enriched in 10-week-old AKC-mice^50^ (Fig. 5f). A recent study performed a mouse to human search for proteomic changes associated with pancreatic tumour development and identified a novel set of genes that robustly discriminated against pancreatic cancer cases from matched controls^51^. We found that this gene set was also over-represented in AKC versus KC mice (Fig. 5f). Collectively, these data suggest that our AKC mouse cohort displays a remarkable number of similarities to the molecular changes previously observed in more aggressive subtypes of human PDAC.

**Figure 6:**
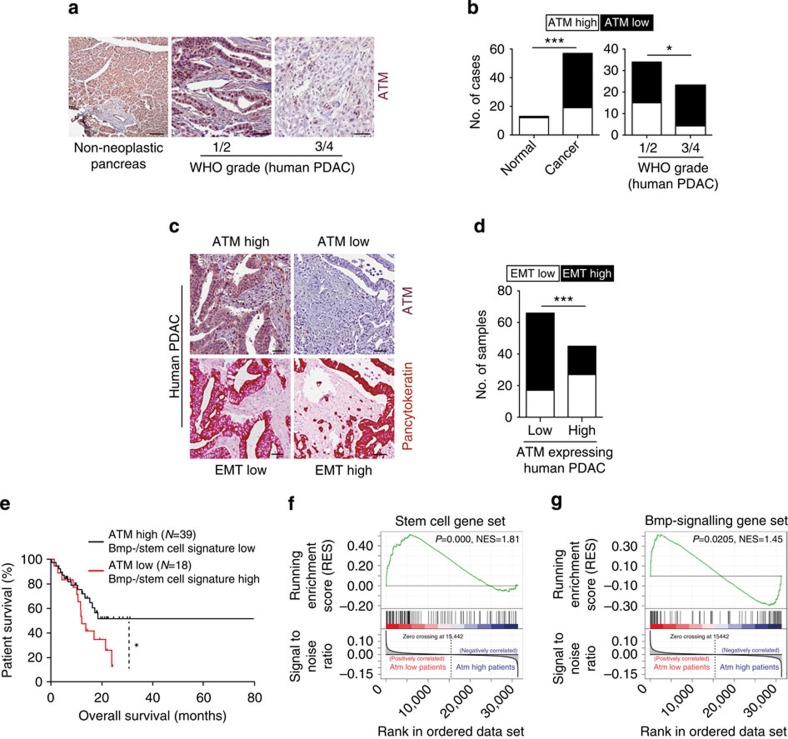
ATM expression in human PDAC. (**a**,**b**) Immunohistochemical staining (**a**) and quantification (**b**) of ATM protein expression in human pancreatic sections. Tumours were graded according to the WHO classification. Numbers analysed are given in the text. Left panel, scale bar, 100 μm; middle and right panel, scale bar, 50 μm. *P* values are determined by two-sided Fisher's exact test. *P=0.0472, ***P=0.0001. (**c**,**d**) The same TMA set from (**a**,**b**) was stained for pancytokeratin. (**c**) Representative images for ATM-low/EMT-high case and vice versa are shown. Scale bar, 50 μm. (**d**) Quantification of all samples for EMT according to ATM status. *P* values are determined by two-sided Fisher's exact test. ***P=0.0004. (**e**) Pancreatic cancer data from ICGC were retrieved from GSE36924. The samples were divided into two, ATM-high (>7.6) and ATM-low (=<7.6), groups on the basis of expression of ATM gene. Kaplan–Meier analysis of survival in patients with PDAC reveals that low levels of ATM mRNA correlate with shortened overall survival. Log-rank (Mantel-Cox) test *P<0.05 (**f**,**g**) Accordingly, GSEA was performed on the ATM high versus ATM low patient cohort to evaluate the significance of pre-defined gene sets, (**f**) a stem cell associated gene set^33^,^34^ and (**g**) a BMP4-signalling signature^42^.

Next we investigated ATM expression using human PDAC tissue microarrays. Analysis of normal pancreatic tissues showed 92% expressed abundant ATM protein levels (*n*=12/13). In 57 PDACs, ATM levels in the neoplastic compartment were high in only 33% (19/57) and low in 66% (38/57) (Fig. 6a,b). Moreover, we identified an inverse correlation between ATM protein expression and World Health Organization (WHO) tumour grading. While high-ATM expression levels were detected in 44% (*n*=15/34) of low-grade carcinomas (WHO Grade I and II), this was the case in just 17% (*n*=4/23) of high-grade tumours (WHO Grade III and IV). This indicates that loss of ATM is associated with a less differentiated tumour phenotype. In addition, ATM-low tumours showed significantly more lymph node metastasis (Supplementary Table 1). Notably, ATM protein expression has previously been shown to correlate significantly with human pancreatic tumour invasion in separate cohorts^17^,^52^, independently complementing our findings.

To determine whether the results obtained from our mouse model were translatable to the human disease, we examined ATM expression levels as a predictor for on-going EMT in human PDAC. Indeed, we found that ATM-low cases had more isolated CK-positive invasive cells. These EMT-like features were present in 74%, whereas the ATM-high cases showed such features in only 40% (Fig. 6c,d). This indicates that loss of ATM expression is associated with a more infiltrative/EMT-like phenotype in human PDAC. On the basis of these findings, we examined ATM mRNA levels in a large cohort of surgically resected primary human PDACs. Intriguingly we found that patients with low levels of ATM (*n*=39) had a significantly reduced survival rate compared with patients with high-ATM levels (*n*=18) (Fig. 6e). Finally, we applied the BMP4 signalling signature used in our mouse model to stratify pancreatic cancer patients, which have been separated according to their ATM expression levels (high vs low). In line with our mouse model, GSEA identified significant enrichment of this signature in the ATM low expressing cohort. Of note, the stem cell signature was also enriched. Thus, we found that low ATM expression is associated with perturbed Bmp4 signalling and this is correlated with shortened survival in human PDAC patients (Fig. 6e–g).

**Figure 7:**
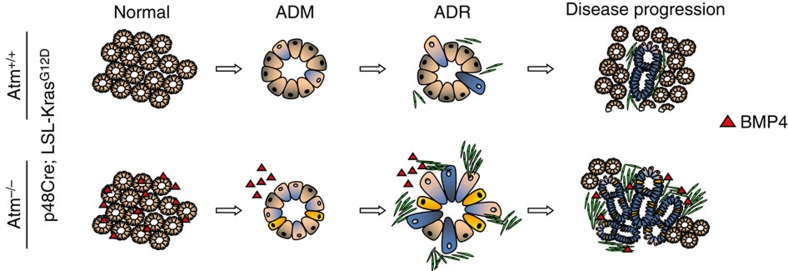
Schematic model depicting the role of ATM in PDAC progression. ATM loss in the context of oncogenic K-ras enhances acinar to ductal reprogramming (ADR) via acinar to ductal metaplasia (ADM) accompanied by EMT and hyperactive BMP4 signalling. Cream colour=acinar structures, blue colour=ductal cells, green=desmoplasia, yellow=ductal cell undergoing EMT.

In summary, our results indicate that deregulation of ATM is a contributing factor supporting PDAC initiation/progression. As shown in Fig. 7, we identified that reduced levels of ATM coupled with oncogenic Kras activation resulted in a higher number of dysplastic pancreatic lesions. This was primarily due to loss of acinar cell identity and a gain in duct-like cell features (acinar-to-ductal metaplasia, ADM), followed by acinar-to-ductal reprogramming (ADR) and PanIN formation, which was broadly associated with an altered TGFβ superfamily signalling and EMT. In addition, these ductal precursor lesions were associated with a pronounced fibrotic reaction. Taken together, our data suggest an intimate link between ATM expression and PDAC progression in mice and men.

## Discussion

ATM is a large serine/threonine kinase implicated in a plethora of cellular functions^9^. Current data suggest that the role of ATM in tumorigenesis is context and tissue dependent. Certain cell types become more sensitive to DNA-double strand break (DSB)-inducing agents upon ATM loss^53^. In contrast, the DNA-damage response acts as an inducible barrier in human gliomas frequently harbouring inactivating mutations in the *Atm/Chk2/Trp53* axis^54^. In the current study, we note that neither members of the DDR pathway including, γH2AX and p21 nor the apoptosis or senescence programmes are remarkably altered in response to loss of ATM. A recent study shows in lung and breast cancer cells that ATM acts as a binary switch to control the contribution of p53 signalling to the DNA damage response and to determine treatment response^55^. Thus, further studies are warranted to investigate the Atm-Trp53 axis in pancreatic cancer in more detail. ATM has previously been shown to prevent dysplastic growth in the colon, independent of its effects on genomic stability^56^. Thus, our data are in line with accumulating evidence that ATM integrates with a variety of signalling cascades and future studies will help unravel the role(s) of ATM apart from its established role in the DDR programme^9^.

ADM in the pancreas is an emerging field of research and recently key transcription factors implicated in this process have been characterized. One such factor, SOX9, accelerates formation of pre-malignant lesions when concomitantly expressed with oncogenic K-RAS^19^. Together with the EMT-promoting factor SLUG, SOX9 determines the mammary stem cell state but also drives EMT. In turn, their co-expression increases tumorigenic and metastatic features of human breast cancer cells and is associated with poor patient survival^26^,^27^. We identify that ATM loss in a mouse model of PDAC enhances SOX9 expression even in intact acinar structures, implicating an intimate connection between ATM and ductal programming. Interestingly, this link seems to be conserved among several cancers as ATM−/− lymphoblastoid cells also show significantly increased SOX9 levels compared with their ATM+/+ counterparts^57^. Ductal programming in ATM-depleted pancreas displayed close correlation with on-going EMT, shown by high levels of ZEB1 and other EMT markers in precursor lesions. Importantly we validated this independently of our microarray analysis by microdissection of neoplastic lesions and sorting for DBA+ cells. Given the large amount of neoplastic lesions in the pancreas at this time point, this is undoubtedly contributing to the microarray data in addition to the fibrosis. Notably, the EMT-TF ZEB1 is a marker for metastatic properties and stemness but also serves as an independent predictor of mortality in PDAC^2^.

ATM loss was recently reported to enhance breast cancer stem cell properties via a TGFβ- dependent mechanism^58^,^59^. In the current study, we found that loss of ATM alters TGF-β superfamily signalling as shown by a perturbed BMP4/SMAD1/5/8 and NODAL/SMAD2/3 signalling axis. Nodal governs EMT via induction of SNAIL and related paracrine signalling events help to establish a niche for tumour initiating cells as shown for pancreatic stellate cells^39^,^60^. Moreover, high NODAL/SMAD2/3 signalling levels in human PDAC cohorts determine poor survival^61^ and an EMT phenotype^37^. Similarly, the BMP4/SMAD1/MMP2 axis drives EMT in PDAC^40^ and notably, our data unravel a series of concomitant events aligned with these published observations. Intriguingly, loss of ATM activity in the pancreas gives rise to elevated BMP4 signalling that acts as a switch in the maintenance of acinar cell integrity and induces trans-differentiation to metaplastic ductal cells. The phenotype observed fits well with previous models in which mice with intact Smad4 signalling display a TGFβ-dependent, EMT-associated tumour growth^62^.

Recent exome-sequencing data from human PDAC and respective progenitor lesions have positioned ATM among the 16 most commonly mutated genes in human PDAC^5^,^15^,^16^. Most recently, ATM mutations were observed predominantly in genetically instable human PDACs^3^ and ATM was one of the few genes already mutated in the germline of PDAC patients^12^. The current study provides molecular insight into the consequences of ATM mutations in PDAC. We highlight key aspects of ATM loss in murine tumour biology and demonstrate that this model faithfully recapitulates subtypes of human PDAC. Low-ATM levels are associated with increased BMP4 levels and elevated stem cell gene signatures, which have been previously linked to disease outcome in a variety of cancers including colorectal cancer^34^. This supports the hypothesis that loss of ATM activity gives rise to more aggressive, EMT-rich tumours due to increased tumour initiating cell potential. Our data uncover that loss of heterozygosity of ATM in AKC mice is sufficient to reduce survival. This observation is in line with the previously reported higher risk of cancer risk and mortality in human ATM heterozygous-mutated patients^23^ and supports previous studies in mice^22^. Also, sequencing analysis of human PDAC cases reported heterozygous mutations in the ATM gene^5^ and linked ATM to BRCA in genetically unstable PDAC subtypes^3^. In line, ATM heterozygosity cooperates with loss of Brca1 to generate anaplastic breast cancer^21^,^63^. It will be interesting to investigate how co-deletion of such factors affects PDAC formation. Taken together our current finding that loss of ATM is implicated in the early stages and progression of PDAC suggests new avenues of signalling mechanisms in PDAC. Therefore, this study facilitates considering ATM as a potential clinical target in human PDAC.

## Methods

### Mouse strains

To generate p48Cre;Kras^G12D/+^;Atm^lox/lox^; we backcrossed the p48Cre and Kras ^G12D/+^ lines^64^,^65^ to the ATM^lox/lox^ line^66^ twice to generate p48Cre; Atm^+/lox^ and Kras^G12D/+^;Atm^+/lox^ mice. We then crossed Kras^G12D^;Atm^+/lox^ mice to p48Cre^+^;Atm^+/lox^ mice to produce experimental animals on a mixed C57/BL/6,129/BALB/c background. Representative genotyping is shown in Supplementary Fig. 1a. Some mice were also bred with Z/AP, a double reporter mice line as previously described^67^ or Rosa26_tdRFP^lox/lox^ mice^30^ to allow for tracing Cre-recombinase expression driven by the p48 promoter (Supplementary Fig. 1b, 4b). Tail-derived DNA was used to confirm the genotype of mice from the breeding crosses using specific primers and representative genotyping is shown in Supplementary Fig. 1a. Murine genotypes followed expected mendelian frequency and primers used for genotyping are outlined in Table 2. All studies were performed under ethical and animal protection regulations of the University of Ulm.

### Histology and Immunohistochemistry

Tissue specimens were fixed in 4% phosphate-buffered paraformaldehyde overnight at 4 °C and embedded in paraffin. Immunohistochemical analyses were performed on serial sections of 4 μm using standard techniques. Antibodies used are outlined in the supplementary Materials and Methods. All images were taken with Olympus BX40 with spot insight QE camera or Mirax Scan (Carl Zeiss), and the IF images were taken with a Zeiss EL-Einsatz Axioskop (Carl Zeiss).

### RNA isolation and Quantitative RT-PCR

RNA was extracted according to the manufacturer's instructions using the RNeasy Mini or Micro Kits (Qiagen) and eluted in 40 or 15 μl RNAse-free H_2_O. cDNA was subsequently synthesized using the iScript cDNA synthesis kit (BioRad). Quantitative real-time RT-PCR analysis (qPCR) was carried out according to the manufacturer's instructions. The PCR reaction was performed using the SensiMix SYBR kit (Bioline) in a Rotor-Gene 6000 series thermal cycler (Qiagen) using the following PCR reaction: denaturation at 95 °C for 2 min, followed by 40 cycles at 95 °C for 15 s, 60 °C for 1 min and 72 °C for 15 s. To verify the specificity of the PCR amplification products, melting curve analysis was performed. mRNA levels were normalized to *Hmbs*, *cyclophilin A or Gapdh* mRNA levels. Primer information is shown in Table 2. Primer sequences are not available for commercial primers from Qiagen.

### Histopathological analysis and scoring of pancreatic lesions

Tumours were scored in blinded manner by a board-certified pathologist (J.K.L.). On the basis of a combination of H&E, Alcian Blue and CK19 stainings, ADM and PanIN lesions were classified according to histopathologic criteria^68^. At least 10 fields at high-power magnification were imaged and characterized for ADM lesions and PanIn stage. For grading of tumour differentiation all tumours were classified by using WHO criteria^69^. Antibody information is shown in Table 3.

### Gene expression microarrays

In brief, total RNA extracted from individual KC and AKC mouse pancreata at 5 (*n*=3 KC; *n*=3 AKC) or 10 (*n*=3 KC; *n*=4 AKC) weeks old was extracted as described above. Genome-wide gene expression profiles of individual AKC and KC samples were generated using the SurePrint G3 Mouse GE 8 × 60 K (Design ID 028005) Microarray Kit (Agilent Technologies). Fifty nanograms of each sample was labelled with the Low Input Quick Amp Kit (Agilent Technologies) according to the manufacturer's instructions. Slides were scanned using a microarray scanner (Agilent Technologies). Raw data were pre-processed and quantile normalized using R/Bioconductor. The expression level of individual gene was then calculated by averaging the signal intensities of all corresponding probe sets. The differential expression analysis of any two conditions was performed using limma *t*-test in R/Bioconductor. Accordingly, the genes that have fold change >1.5 and corrected *P* value <0.05 were considered as being differentially expressed. Array data are available in GEO under accession code GSE68808. To gain functional insight of differentially expressed genes, the enrichment analysis was conducted using pre-defined gene sets^28^,^29^,^33^,^42^,^50^,^51^ and customized R script of GSEA. Human PDA tumour samples were retrieved from GSE17891 (39 samples) and GSE17891 (26 PDA samples) in NCBI GEO. To compare ATM samples and human PDA in Fig. 5e and minimize the batch effect, all samples were subjected to COMBAT algorithm, resulting in a merged data set including 73 samples and 14,468 genes. Hierarchical clustering was performed in *R*. In Fig. 6e–g, pancreatic cancer data from ICGC were retrieved from GSE36924. The samples were divided into two, ATM-high (>7.6) and ATM-low (=< 7.6), groups on the basis of expression of *ATM* gene. Accordingly, the GSEA was performed on the data set to evaluate the significance of pre-defined gene sets^33^,^42^.

### Tissue microarrays

Commercially available tissue microarray sections of human PDACs (A207IV and A207V AccuMax Array) were purchased from ISU ABXIS (Seoul, Korea) and used according to the manufacturer's instructions (The human biological product provided was obtained legally, in compliance with applicable national and local laws, regulations and guidelines.). In brief, after deparaffinization, antigen retrieval was performed in Sodium Citrate solution (pH 6). Sections were incubated with rabbit anti-ATM (#sc-7230, Santa Cruz at 1:1,000 dilution) or Cytokeratin (DAKO, M3515; clone AE1+AE3; at 1:100 dilution with pronase pretreatment) for 1 h at RT. Sections were then washed and incubated with secondary antibodies for 45 min at room temperature. For visualization, 3,3′diaminobenzidine tetrahydrochloride substrate (DAB) was used as a substrate and sections were lightly counterstained with haematoxylin, dehydrated and mounted. Nuclear ATM staining was assessed in neoplastic epithelial cells and a case was scored as high when there was >10% labelling within the tumour cell fraction and <10% positive tumour cells were scored as low. The entire neoplastic population was visualized by pancytokeratin staining.

### Western blot analysis

Immunoblotting was performed according to standard procedures. In brief, a piece of pancreas was frozen in liquid nitrogen immediately after killing the mice. Protein lysates were prepared using protein extraction buffer (4% SDS, 100 mM Tris-HCl) containing protease inhibitors and 1 mM phenylmethylsulfonyl fluoride (PMSF), and cell debris was removed by centrifugation at 4 °C for 10 min at 14,000 r.p.m. Protein content was measured using a colorimetric assay (Bradford Biorad Assay, Biorad). Lysates (30 μg) were resolved by SDS–PAGE and transferred to a PVDF membrane (#IPVH00010, Immobilon-P Membrane, Millipore). Immunoreactive bands were visualized using chemiluminescence (Thermo scientific, Waltham, MA, USA) (Supplementary Fig. 6). Images were processed and analysed using the ImageJ software (http://rsbweb.nih.gov/ij/). Antibodies used are as follows: goat anti-Bmp4 (#sc-6896 Santa Cruz); rabbit anti-Nodal (#39953, Abcam) rabbit anti-Phospho Smad 1/5/8 (#9511, Cell Signaling); rabbit anti-Phospho Smad 2/3 (#3101, Cell Signaling); rabbit anti-ATM (#ab78, Abcam) all 1:1000, O.N. at 4° and mouse anti-β-actin (#3101, Sigma) 1:50,000 for 1 h at RT.

### Microdissection of ADM and PanIN lesions

Paraffin-embedded mouse tissues were sequentially sectioned in three to four 10-μm-thick sections and a single 3-μm-thick section and were mounted onto glass slides. Sections were then deparaffinized in xylene, rehydrated using graded ethanol and washed with distilled water. The 3-μm-thick section was stained in haematoxylin and eosin and coated with glass slides to properly assess the morphology of the progenitor lesions. The 10-μm-thick slides were stained in eosin only and were then transferred to manual microdissection. Within each slide ADM and PanIN lesions were dissected separately. RNA isolation was performed utilizing the Qiagen miRNeasy FFPE Kit following the manufacturer's instruction, and cDNA synthesis was done using 150-200 ng of RNA. The samples were subjected to qRT-PCR as described above. EMT marker expression was normalized to *Ck19* expression levels, and Mann–Whitney test was performed to determine statistical significance.

### Acinar cell isolation and culture

Acinar cell explants were isolated and cultured as previously described with slight modification^70^. In brief, the pancreas of mice aged 4-6 weeks was removed and placed in cold Hank's Balanced Salt Solution (HBSS). The pancreas were then chopped into small pieces and then transferred to Collagenase P solution (0.2 mg ml^−1^) for ∼10 min at 37 °C. The digested pancreas was washed twice with HBSS containing 5% FCS and filtered through a 100-μm cell strainer (Greiner bio-one). The tissue suspension was then layered gently on a 30% FCS/HBSS solution and centrifuged at 4 °C to pellet the acini. The cell pellet was then resuspended in a 1:2 media: growth factor reduced matrigel Beacton Dickinson (BD) solution and plated on a 24-well-cell culture plate. Media used for culturing the cells was Waymouth's, 10% FCS, 1% P/S, 1 mg ml^−1^ dexamethasone (Sigma) and 100 mg ml^−1^ Soybean trypsin inhibitor (Sigma). Media was changed daily and human recombinant BMP4 (Preprotech 120-05) and human recombinant Noggin (Preprotech 120-10C) were used at 25 ng ml^−1^ and 5 μM, respectively. Dorsomorphin and LDN-193189 (Sigma) were used at concentrations as indicated in Fig. 4h–j and Supplementary Fig. 5 (ref. 43). RNA was isolated at day 2 and PCR analysis was performed as described above. Quantification of ductal structures was performed by counting at least six individual fields at × 10 magnification in triplicate.

### Statistical analysis

Contingency graph statistics were calculated using the Fisher exact test. All other tests for significance, unless otherwise stated, were performed using an unpaired Student's *t*-test. Kaplan–Meier curves were calculated using the survival time for each mouse from all littermate groups. The log-rank (Mantel-Cox) test was used to test for significance differences between the groups. Statistical analysis was performed using GraphPad Prism 5 (GraphPad Software). Error bars represent the s.e.m.

### Proliferation index

The proliferation index for ADMs and mPanINs was determined by counting the Ki67-positive nuclei (actively proliferating cells) per field for 10 fields at × 40 magnification, with at least four mice per group. Each field selected contained ADMs and mPanINs exclusively and if stroma cells were within the field, these were excluded from the count. Differences between groups were evaluated using the Student's *t*-test, where *P*<0.05 was considered to be significant.

### Fibrotic content

Sections were stained with Masson–Goldner stain and histologic fibrosis was evaluated in at least four mice per group. Images of each section were recorded at × 2.5 magnification and fibrotic regions were defined as distinct blue/grey areas. The total area of each fibrotic region was measured and expressed as a percentage of the total section area. All measurements were recorded and analysed using Image J software. Differences between groups were evaluated using the Student's *t*-test, where *P*<0.05 was considered to be significant.

### Senescence-associated-β-galactosidase (SA-β-gal) assay

Frozen sections of pancreatic tissue were fixed with 4% paraformaldehyde in PBS for 15 min, washed with PBS and stained at 37 °C in the dark for 14–16 h in X-Gal solution (1 mg ml^−1^ X-Gal, 40 mM Citric-acid Sodiumphosphate, 5 mM potassium ferrocyanide, 5 mM potassium ferricyanide and 1 mM MgCl2 in PBS at pH 5.3) and subsequently counterstained with Hoechst.

### Cytokine antibody array

Proteins present within pancreata form the respective genotypes were screened using the RayBio Mouse Cytokine Antibody Array C series 1000 (RayBiotech) according to the manufacturer's instructions. Total pancreata from a p48Cre;Kras^G12D/+^;Atm^+/+^ and p48^Cre/+^;Kras^G12D/+^;Atm^−/−^ mouse were lysed in 1 × Lysis Buffer and cleared via centrifugation. The amount of lysate used was normalized to protein content. The membranes were incubated with the cleared lysates, washed and incubated with the provided primary biotin-conjugated antibody. After subsequent washes, the membranes were incubated with horseradish peroxidase-conjugated streptavidin and the protein spots were detected using chemiluminescence (Thermo scientific, Waltham, MA, USA). The spot density was quantified using ImageJ software (http://rsbweb.nih.gov/ij/) and compared between the samples, relative to internal controls.

### Co-immunofluorescence for RFP and Zeb1

Pancreatic specimens were fixed in 4% phosphate-buffered paraformaldehyde overnight at 4 °C and embedded in paraffin. Immunohistochemical analyses were performed on serial sections of 4 μm using standard techniques. In brief, after deparaffinization, antigen retrieval was performed in Citrate buffer (pH 6). Slides were blocked with CAS-Block histochemical reagent (Invitrogen) for 45 min at RT. Anti-Zeb1 primary antibody was used at 1:100 dilution in blocking solution overnight at 4 °C. Sections were then washed and incubated with secondary antibodies for 45 min at room temperature. Subsequently, biotinylated anti-RFP primary antibody diluted 1:200 in blocking solution was added to the sections for 1 h at RT. After washing Vectastain ABC-AP complex (Vector laboratories) was directly applied for 30 min at RT and developed using fluorescence Vector Red Alkaline Phosphatase (AP) Substrate Kit (Vector Laboratories). Controls using each antibody combination alone on serial sections were performed. All IF images were taken using a Zeiss EL- Einsatz Axioskop (Carl Zeiss).

### ATM mRNA expression in human PDAC

In addition to newly generated data, we curated the freely available data portal of the International Cancer Genome Consortium (ICGC; www.icgc.org; last accessed 20 September 2013). In brief, we extracted patient characteristics, clinicopathological data (stage, grade and so on) and outcome data for survival analysis. The associated gene expression data (GSE36924) were extracted from GEO (http://www.ncbi.nlm.nih.gov/geo/; last accessed 20 September 2013). We analysed the frequency distribution of values from the ATM probeset (ILMN_1716231) using a non-linear, fourth polynomic fit. The local maximum (>7.6) was defined as the ATM cutoff for distinction of high versus low ATM.

### Pancreatic ductal cell isolation

Pancreatic ductal cells were isolated according to a recently published protocol^31^. In brief, the pancreas of mice aged 9-12 weeks were removed and placed in G-solution (Hank's Balanced Salt Solution (HBSS), 0.9 g l^−1^ glucose and 47.6 μM CaCL2). After washing, the pancreas were minced into small pieces (< 1 mm^3^) using surgical scissors and scalpels. The tissue pieces were then transferred to DMEM/F12 containing 1 mg ml^−1^ collagenase V (Sigma) and 100 mg ml^−1^ Soybean trypsin inhibitor (Sigma) and incubated at 37 °C for∼35 min with rotation. The reaction was stopped by adding cold G-solution, and the cell suspension was then centrifuged at 300 g for 5 min at 4 °C. The cellular pellet was re-suspended in 2 ml trypsin-EDTA for 2 min at RT using a 1 ml pipette. The reaction was stopped and the cells were pelleted via centrifugation as above. The cellular pellet was subsequently washed with cold separation buffer (PBS, 0.5% BSA and 2 mM EDTA), filtered through a cell strainer and re-centrifuged. The cellular pellet was then separated into 400 μl aliquots and one was kept as a pre-sorting fraction. The other aliquots were subject to staining with DBA lectin-FITC (Vector Laboratories) at a dilution of 1:400 for 10 min on a rotor in the dark at 4 °C. Following washing in the same buffer, cells were centrifuged at 300 g for 10 min. The cellular pellet was then resuspended in 90 μl separation buffer and 10 μl of anti-FITC Microbeads (Miltenyi Biotec) was added and the solution was incubated in the dark for 15 min at 4 °C. After a final washing step, separation was performed with MS columns (Miltenyi Biotec), according to the manufacturer's protocol. RNA from all fractions (DBA-positive, DBA-negative and presorting) was isolated using the RNeasy microkit (Qiagen) and subject to cDNA synthesis as described above. Efficient separation of the different fractions was confirmed using DBA-specific primers (data not shown).

## Additional information

**Accession codes**: Array data are available in GEO under accession code GSE68808

**How to cite this article:** Russell, R. *et al*. Loss of ATM accelerates pancreatic cancer formation and epithelial–mesenchymal transition. *Nat. Commun.* 6:7677 doi: 10.1038/ncomms8677 (2015).

## Supplementary Material

Supplementary Information Supplementary Figures 1-6, Supplementary Table 1 and Supplementary References

## Figures and Tables

**Table 1:** ATM genetic alterations in human PDAC.

**ICGC data set**	**Number of donors harbouring** ***ATM*** mutations*	**Number of donors in data set**	**Per cent**
PACA-AU	37	392	9.4%
PACA-CA	36	199	18.1%
Total	**73**	**591**	**12%**

ATM=ataxia telangiectasia-mutated; AU=Australia; CA=Canada; ICGC=International Cancer Genome Consortium (last accessioned: 3/24/2015); PACA=pancreatic cancer; PDAC=Pancreatic ductal adenocarcinoma. Mutation frequency of ATM mutations in PDAC. Table showing the frequency of ATM mutations in two independent cohorts of PDAC patients. Data extracted from the ICGC database.

^*^Non-synonymous substitutions or indels.

**Table 2:** Primers used for genotyping and qPCR analysis.

**Primers**	**Company**	**Forward sequence**	**Reverse sequence**
***Genotyping***			
Cre001	Biomers	5′-accagccagctatcaactcg-3′	
Cre002	Biomers		5′-ttacattggtccagccacc-3′
Kras005	Biomers	5′-cctttacaagcgcacgcagactgtaga-3′	
Kras006	Biomers		5′-agctagccaccatggcttgagtaagtctgca-3′
ATM gF86723	Biomers	5′-atcaaatgtaaaggcggcttc-3′	
ATM BAC13	Biomers	5′-catcctttaatgtgcctcccttcgcc-3′	
ATM BAC 7	Biomers		5′-gcccatcccgtccacaatatctctgc-3′
RosaRFP1 (SH57A)	Biomers	5′-gcaatctatacatgtcctgcgag-3′	
RosaRFP1 (SH65A)	Biomers	5′-catacgtgtatatgatgggaggttg-3′	
RosaRFP1 (LO86)	Biomers		5′-ggtcacgagggtgggcca-3′
			
***qPCR***			
Amylase	Biomers	5′-cagagacatggtgacaaggtg-3′	5′-atcgttaaagtcccaagcaga-3′
CK19	Biomers	5′-gggggttcagtaattgg-3′	5′-gaggacgaggtcacgaagc-3′
Sox9	Biomers	5′-cttctgtgggagcgacaactt-3′	5′-agggagggaaaacagagaacg-3′
Cyclophilin A	Biomers	5′-ccctccacccatttgct-3′	5′-caatccagctaggcatggga-3′
Vimentin	Biomers	5′-caggccagattcaggaaca-3′	5′-acgctttcatactgctggcg-3′
alpha SMA	Biomers	5′-cgctgctccagctatgtgtgaa-3′	5′-ccctgggagcatcatcacc-3′
Slug	Biomers	5′-agatgcacattcgaacccac-3′	5′-gtctgcagatgagccctcag-3′
DBA	Biomers	5′-caatctccccttcggatacc-3′	5′-cacatcagcaagggtgttca-3′
FSP1	Biomers	5′-ggagctgcctagcttcc-3′	5′-ctgtccaagttgctcatca-3′
N-cadherin	Biomers	5′-catcaaccggcttaatggtg-3′	5′-actttcacacgcaggatgga-3′
Fibronectin	Biomers	5′-tacacctgctcctgtgcttc-3′	5′-gagacctgctcctgtgcttc-3′
			
***Microdissection qPCR***			
CK19	Biomers	5′-cctcccgcgattacaaccact-3′	5′-ggcgagcattgtcaatctgt-3′
Sox9	Biomers	5′-cgtgcagcacaagaaagacca-3′	5′-gcagcgccttgaagatagcat-3′
N-cadherin	Biomers	5′-tgggtcatcccgccaatcaa-3′	5′-aaccgggctatcagctctcg-3′
Slug	Biomers	5′-gctttctccagaccctggct-3′	5′-tgcagatgtgccctcaggtt-3′

**Table 3:** Antibodies and dyes used for histological evaluations.Abbreviation: NA=not applicable.

**Antibodies:**	**Company:**	**Catalogue No.**	**Species**	**Dilution IHC**	**Dilution IF**
***Primary antibodies***					
Collagen 1	Abcam	ab34710	Rabbit	1:500	
Zeb1	Santa Cruz	sc-25388	Rabbit	1:100	1: 2,000
Sox9	Millipore	AB5535	Rabbit	1:200	1: 8,000
α-SMA	Biozol	AB5694	Rabbit	1:400	
Amylase	Sigma	A8237	Rabbit	1:50	1:100
N-cadherin	Zymed	33-3900	Mouse	1:100	
CK19	Abcam	ab7755	mouse	1:100	1: 2,000
Cyclin D1	Santa Cruz	sc-753	Rabbit	1:50	
Cyclin E	Santa Cruz	sc-481	Rabbit	1:200	
BMP4	Santa Cruz	sc-6896	Goat	1:200	
Fibronectin	Biomol	A103	Rabbit	1:200	
p53	Abcam	ab26	Mouse	1:250	
ATM	Santa Cruz	sc-7230	Rabbit	1:100	
CPA1	R+D Systems	AF2765	Goat	1:100	1: 5,000
ki67	Dako	M7249	Rat	1:100	
CD45R	BD	550286	Rat	1:50	
Vimentin	Max Plank Institute	NA	Mouse	1:50	
CD133	Miltenyl Biotec	130-090-422	Mouse	1:20	1:20
Ck8/18	Progen Biotechnik	GP11	Guinea pig	1:200	
RFP	Abcam	ab34771	Rabbit		1:100
					
***Secondary antibodies***					
Alexa Fluor 488	Life Technologies	A-11001	Mouse		1:1,000
Alexa Fluor 488	Life Technologies	A-11008	Rabbit		1:1,000
Alexa Fluor 568	Life Technologies	A-10037	Mouse		1:1,000
Alexa Fluor 568	Life Technologies	A-11057	Goat		1:1,000
Anti-guinea pig IgG (H+L)	Vector	BA7000		1:100	
Anti-rabbit IgG (H+L)	Vector	BA-1000		1:100	
Anti-mouse IgG (H+L)	Vector	BA-2000		1:100	
Anti-goat IgG (H+L)	Vector	BA-5000		1:100	

**Dyes**	**Company:**	**Catalogue No.**
Tunel	Calbiochem	Q1A33
Masson Goldner	Merck	100485
Alcian blue	NA	NA
