# Supplementary material for: Loss of ATM accelerates pancreatic cancer formation and epithelial–mesenchymal transition
Source: Nat Commun. 2015 Jul 29;6:7677. doi: 10.1038/ncomms8677 (PMC4532798; doi:10.1038/ncomms8677)
Supplement: Supplementary Information — Supplementary Figures 1-6, Supplementary Table 1 and Supplementary References [file ncomms8677-s1.pdf]

Supplementary Figure 1

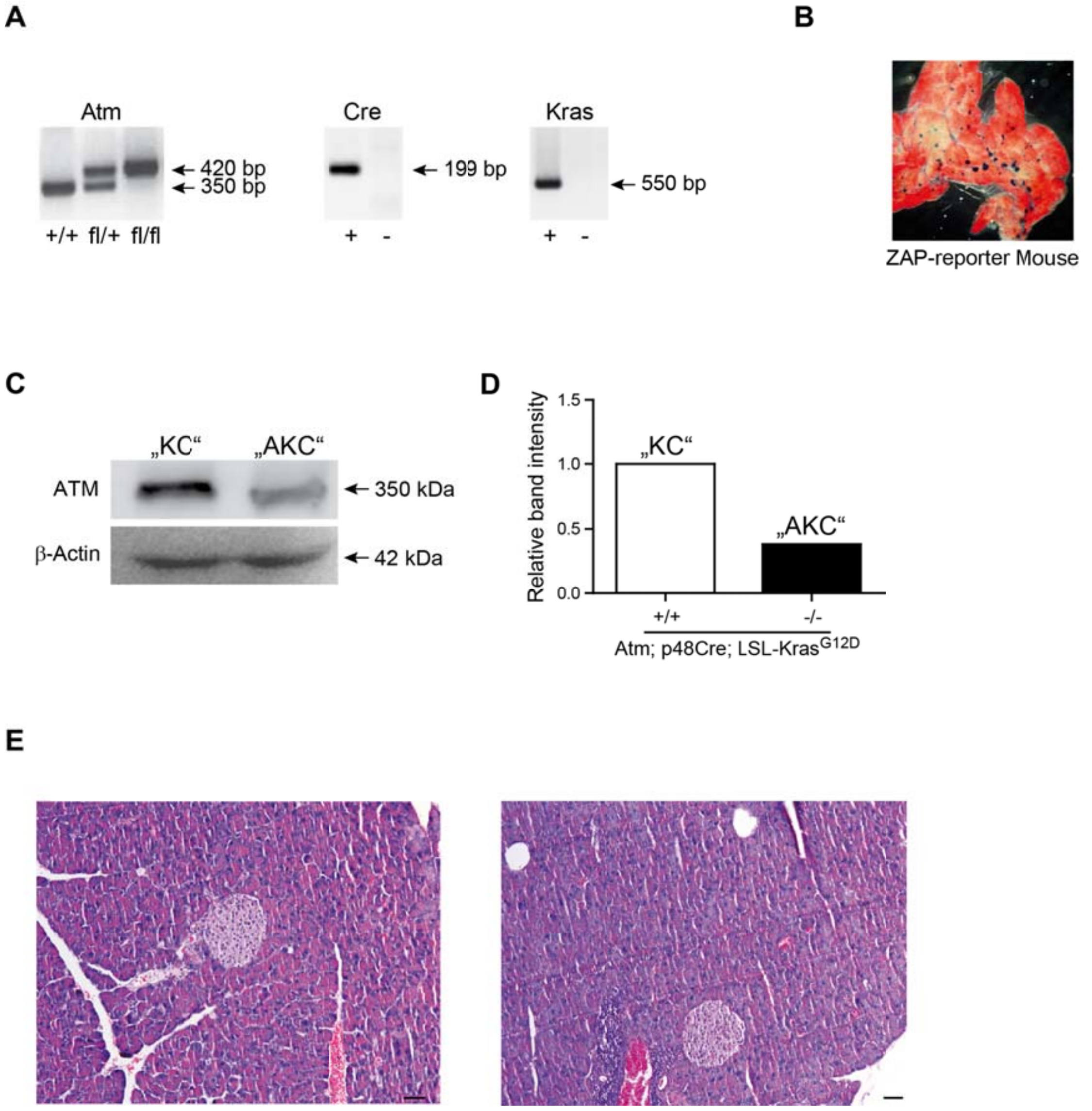

Supplementary Figure 1. Characterization of the conditional ATM mouse model co-expressing oncogenic K-RAS. (**A**) Genotyping PCR from tail DNA shows expression of ATM wildtype and floxed alleles, p48Cre recombinase and $Kras^{G12D}$ with the respective negative controls. (**B**) Representative image of the pancreas from Z/AP-reporter mouse[1] in which p48Cre- activation results in a homogenous AP (in red) reporter signal indicating almost complete recombination within the exocrine compartment of the mouse pancreas. Blue staining delineates non-recombined islets. (**C,D**) Representative immunoblot and respective quantification of ATM expression in the pancreas of mice from the indicated genotypes. (**E**)

Representative images of the pancreata from p48Cre;$Atm^{-/-}$ mice pancreas over 1 year of age reveal normal pancreatic architecture.

Supplementary Figure 2

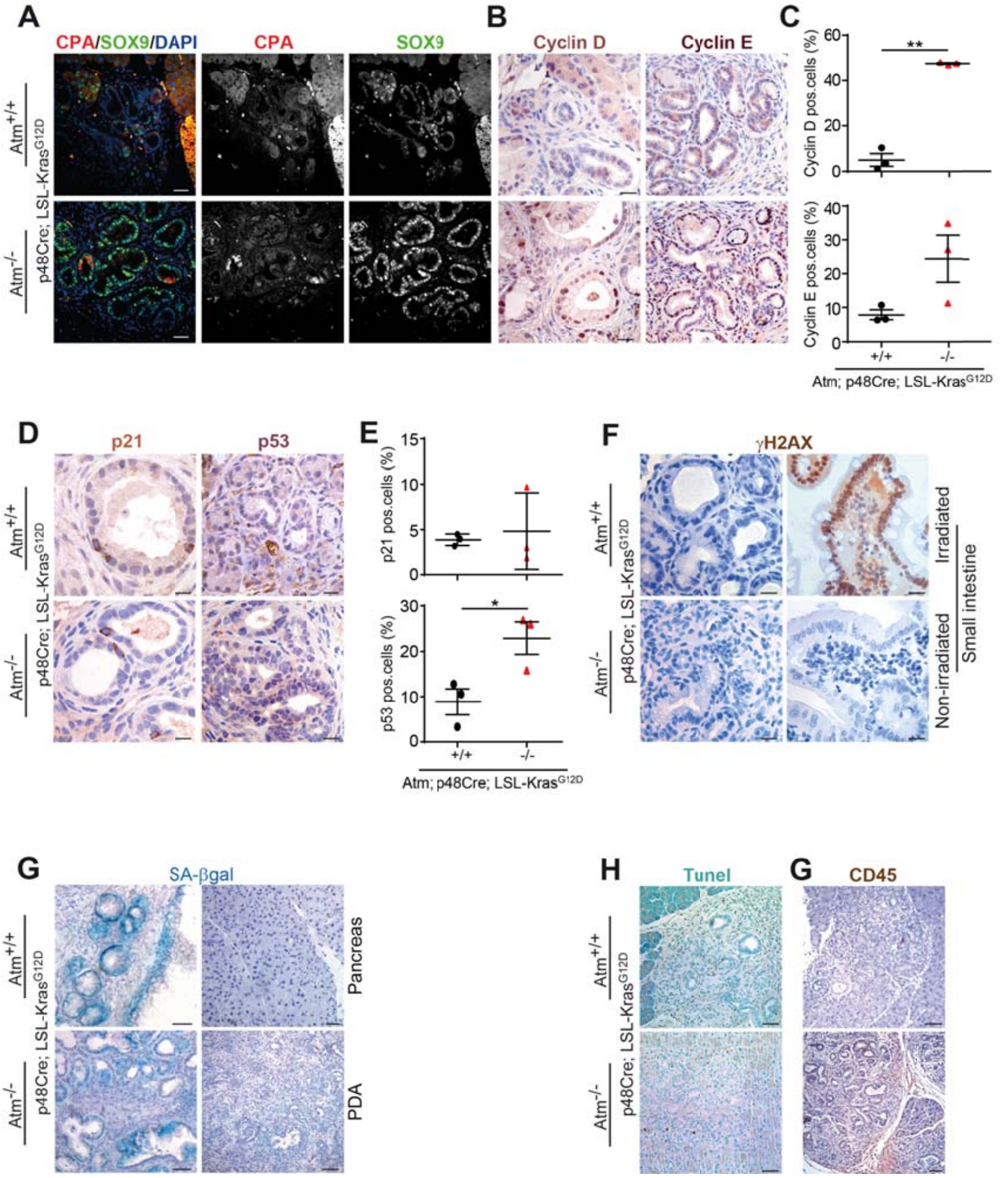

Supplementary Figure 2. Immunohistochemical analysis of KC- and AKC mice for different markers. (**A**) Immunofluorescence staining of pancreata from the respective genotypes at 10 weeks old shows expression of CPA (red), Sox9 (green) and Nuclei (Dapi-blue) in precursor lesions. Scale bar, 20 µm . (**B-G**) Immunohistochemical staining reveals (**B**) Cyclin E (Scale bar, 10 µm) and Cyclin D (Scale bar, 20 µm), (**C**) p53 and p21(Scale bar, 10 µm), (**D**) γH2AX (Scale bar, 10 µm), **(E**) SA-βgal (Scale bar, 10 µm; PDA image Scale bar

10 μm), (**F**) Tunnel and (**G**) CD45 expression in the respective genotypes and conditions (Scale bar, 20 μm). Irradiated and non-irradiated small intestines from mouse serve as positive and negative controls for γH2AX staining. Normal adult pancreas and PDAC serve as negative controls for SA-βgal. Representative images from at least 3 mice per group are shown. *P<0.05, **P<0.01, ***P<0.0001 (Student's t-test). Error bars, s.e.m.

Supplementary Figure 3

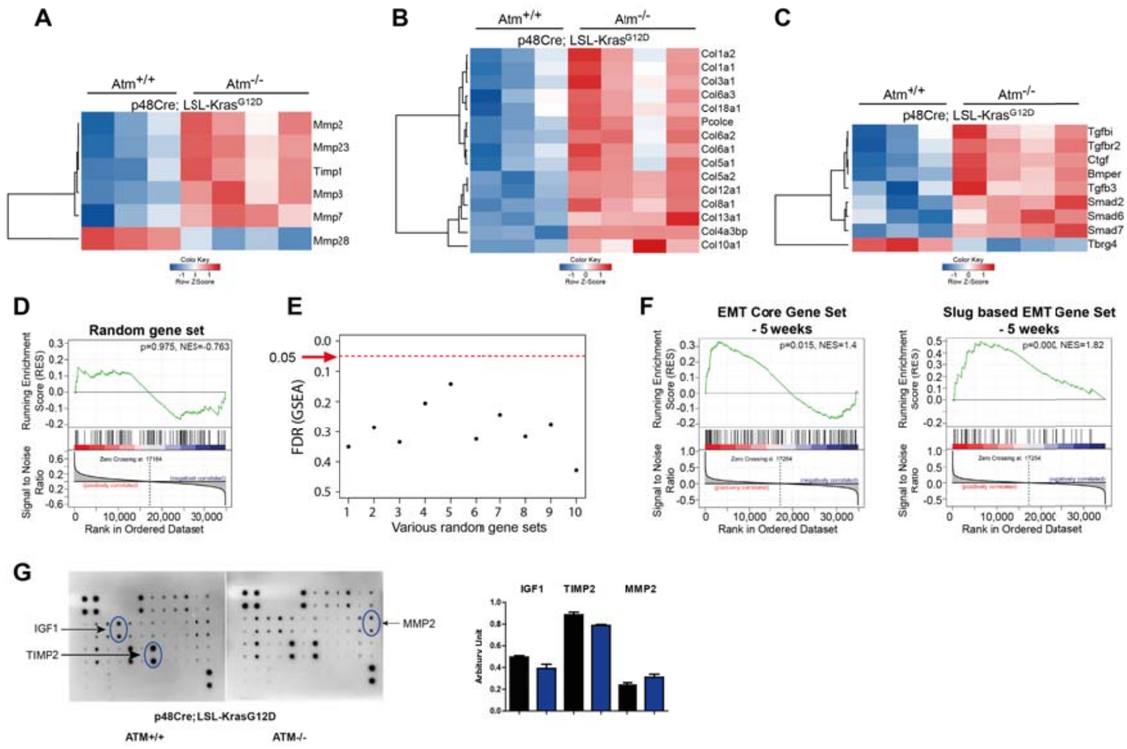

Supplementary Figure 3. Heat map analysis and GSEA of microarray data on AKC and KC mice (**A,B**) Hierarchically clustered heat map illustration a differential expression pattern for numerous genes from the (**A**) MMP and (**B**) collagen families in pancreata from 10 week AKC- vs. KC-mice. (**C**) Hierarchically clustered heat map illustration shows differential expression of TGF-β superfamily members among AKC- and KC-mice. (**D**) A representative GSEA from 10 random gene sets each containing 100 genes randomly selected from the mouse genome shown in **(E)**, shows no enrichment to the differentially regulated gene list shown in Figure 2A. All the p-values are above 0.05, suggesting no similarity or bias to any group of samples. **(F)** Gene set enrichment analysis of differentially regulated genes from **Figure 2A** identifies enrichment of the (**E**) EMT core gene set and slug based EMT gene set (gene sets taken from [2,3]) in AKC - pancreata at 5 weeks of age. (G) A mouse cytokine array using total pancreatic lysate from a p48Cre;Kras$^{G12D/+}$;Atm$^{+/+}$ (left) and a p48$^{Cre/+}$;Kras$^{G12D/+}$;Atm$^{-/-}$ (right) mouse. Error bars, s.e.m.

Supplementary Figure 4

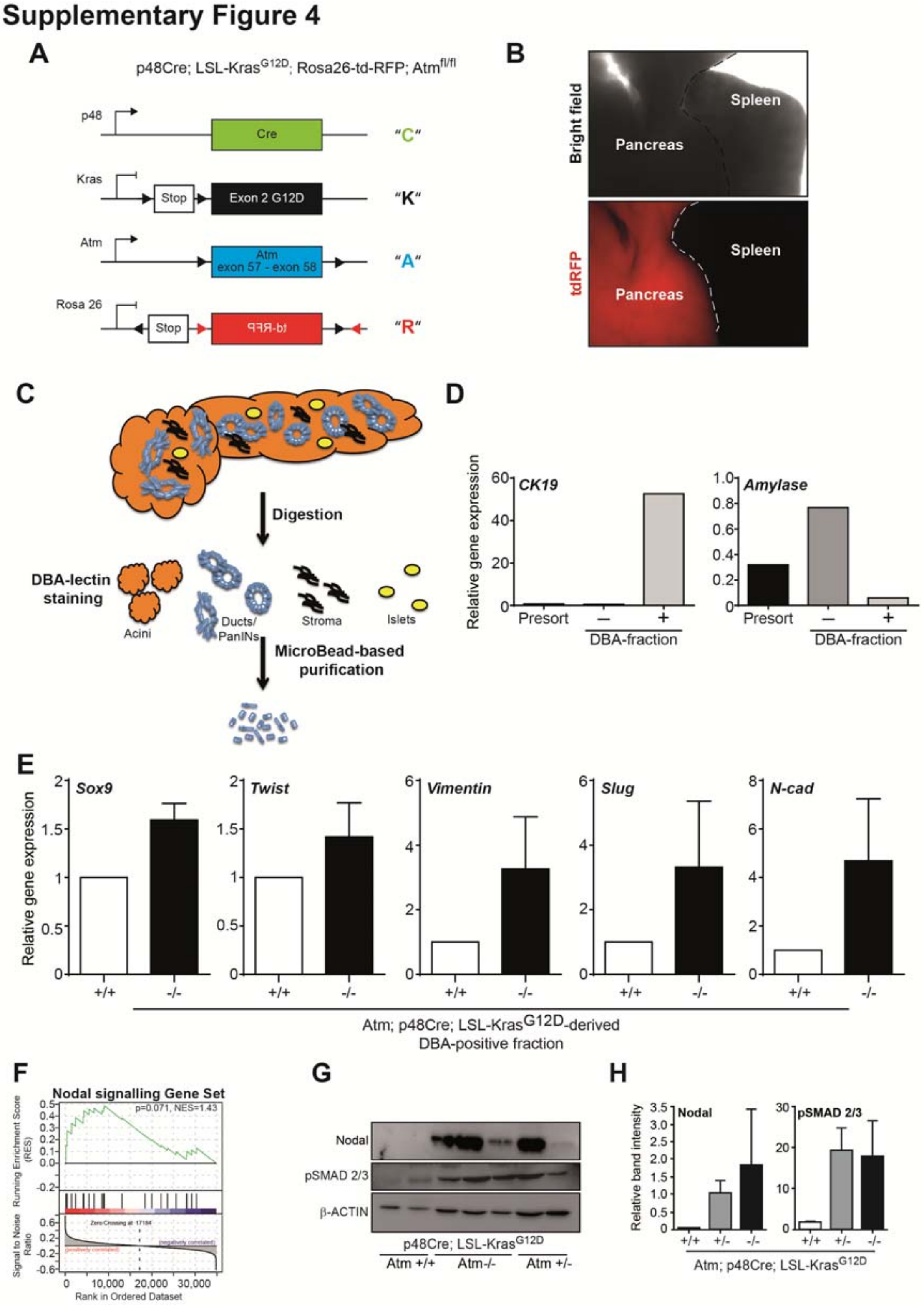

Supplementary Figure 4. ATM loss drives EMT and stemness (**A,B**) Illustration of strategy to generate p48Cre;Kras$^{G12D/+}$;Atm$^{-/-}$;Rosa_tdRFP$^{fl/fl}$ mice (p48$^{Cre/+}$ = "C"; Kras $^{G12D/+}$ = "K"; Atm$^{-/-}$ = "A"; Rosa_tdRFP$^{fl/fl}$ = "**R**") and tdRFP expression in recombined adult pancreas with

no expression found in the spleen. (**C**) Illustrative overview of the DBA-lectin sorting strategy. (**D**) RT-qPCR expression for *Ck19* and *amylase* in the indicated cellular compartment following DBA-lectin MACS sorting. **(E)** RT-qPCR for *Sox9*, *twist1*, *vimentin*, *Slug* and *N-cadherin* in the DBA+ fractions from $p48^{Cre/+}$;$Kras^{G12D/+}$;$Atm^{-/-}$ and p48Cre;$Kras^{G12D/+}$;$Atm^{+/+}$ mice (n=3 per genotype). Error bars, s.e.m. (**F**) Gene set enrichment analysis of differentially regulated genes (from (**Figure 2A**) using previously described gene sets [4] identifies enrichment of the Nodal signalling pathway in AKC pancreata. (**I,J**) Immunoblot and quantification of Nodal, Phospho-Smad 2/3 and β-actin in the respective genotypes. Error bars, s.e.m.

Supplementary Figure 5

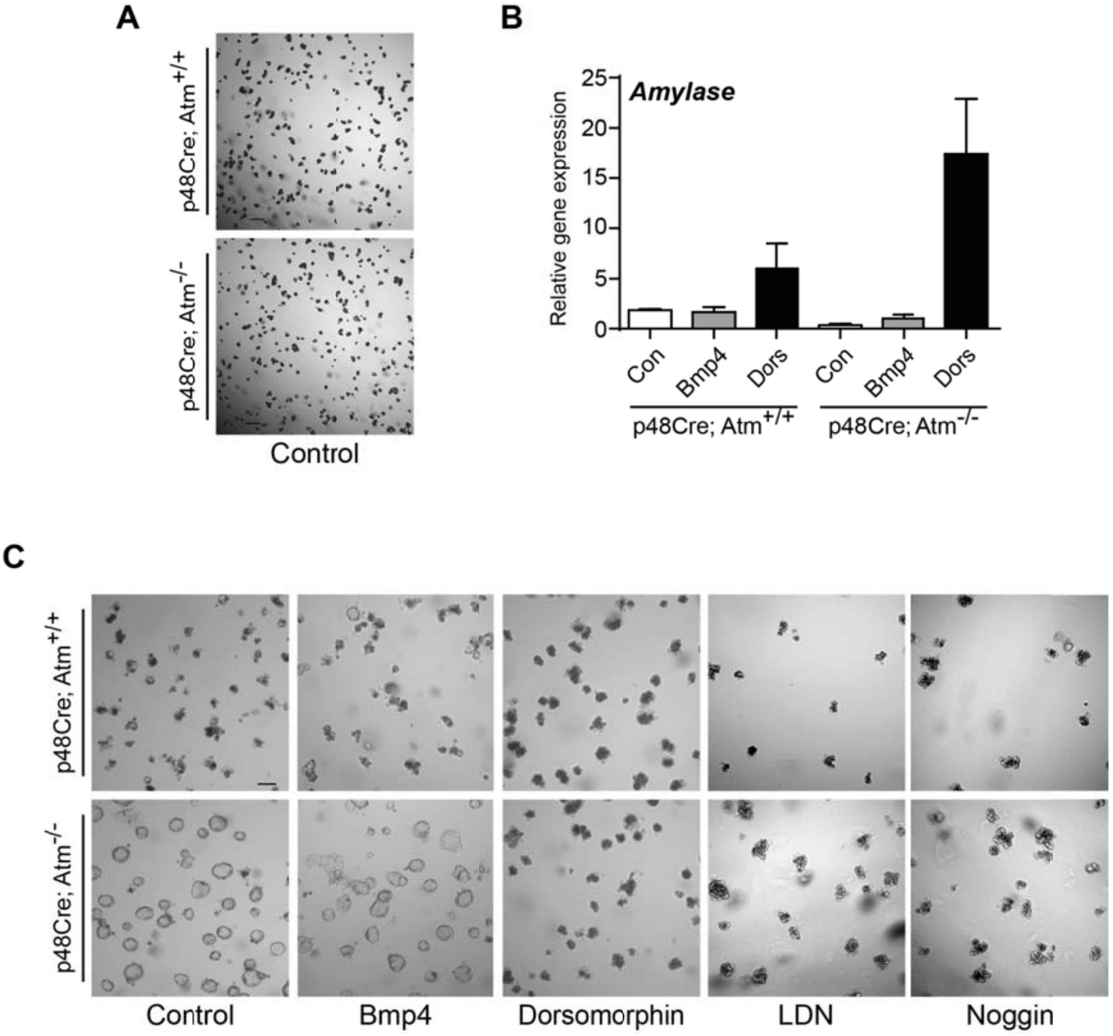

Supplementary Figure 5. Loss of ATM increases ADM formation. (A) Brightfield images of freshly isolated acinar cell cultured at 1 day from the indicated genotypes. Scale bar, 100 µm **(B)** RT-qPCR showing levels of the acinar differentiation marker – *Amylase* in the respective cell culture conditions at day 2. Error bars, s.e.m. (**C**) Low power brightfield (10 x magnification) images of acinar cell cultures under the indicated conditions at day 2 of culture. Scale bar, 50 µm.

Supplementary Figure 6

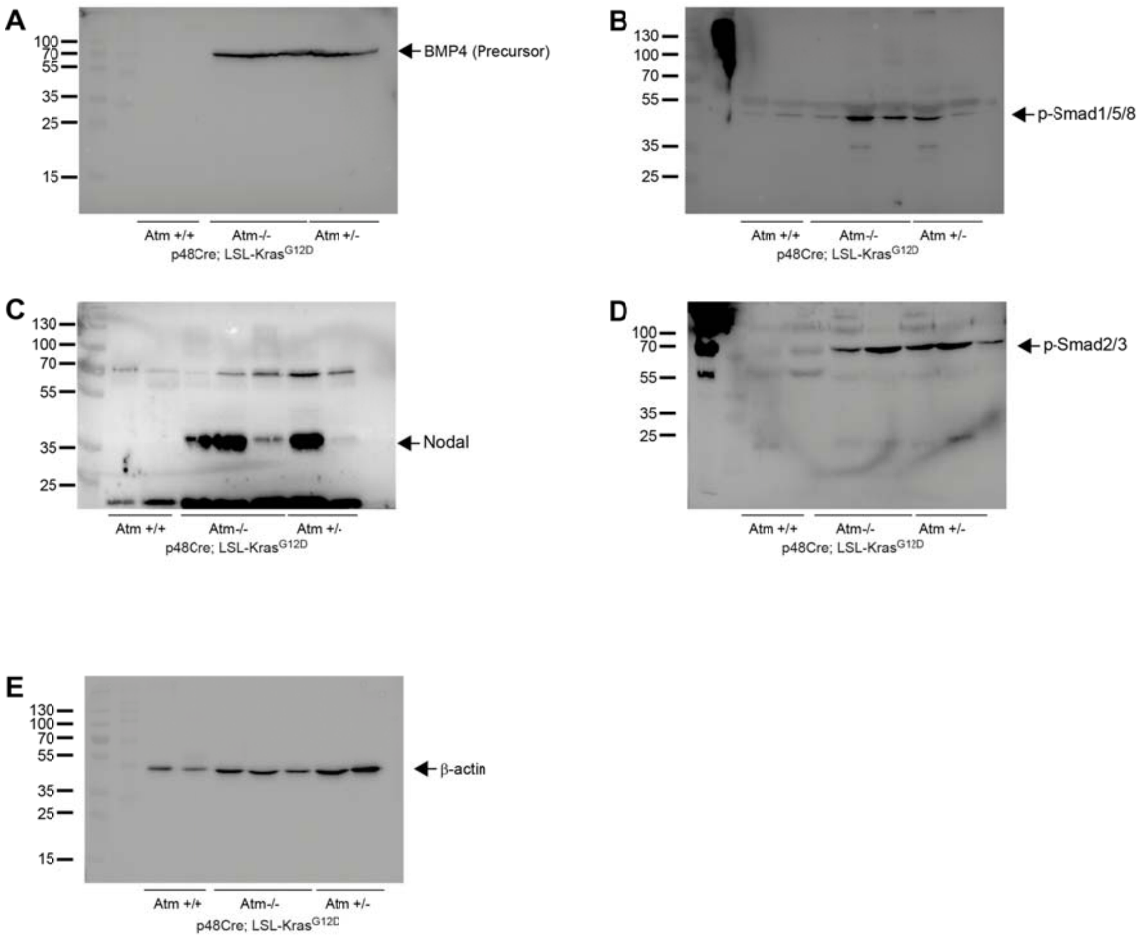

Supplementary Figure 6. Original gel pictures for western blots.

The figure shows uncropped western blots displayed in Fig. 4B and Supplementary Figure 4G. (A,B,C) Western blot analysis of protein levels of BMP4, phosphorylated-Smad1/5/8 and β-actin in whole pancreatic lysates from the indicated groups. (D,E) Western blot analysis of protein levels of Nodal and phosphorylated-Smad2/3 in whole pancreatic lysates from the indicated groups.

Supplementary Table 1. Pancreatic Cancer Cohort (commercial TMA)

**Characteristic**	**All** N=57	%	**ATM-high*** N=19	%	**ATM-low*** N=38	%	***P*** pos vs. neg.
**Sex**							*0.39*
Male	35	61	10	53	25	66	
Female	22	39	9	47	13	34	
**Age**							*0.25*
Median	61		63		61		
(Range)	(32-80)		(45-78)		(32-80)		
**Location**							*0.56*
Head	38	22	12	21	26	24	
Body	13	67	3	63	9	68	
Tail	6	11	3	16	3	8	
**Size**							*1.0*
≤2cm	6	10	2	10	4	10	
>2cm	51	90	17	90	34	90	
**T-Category**							*0.78*
T1	-	-	-	-	-	-	
T2	5	9	2	11	3	8	
T3	47	82	16	84	31	82	
T4	5	9	1	5	4	10	
**N-Category**							***0.01***
N0	25	44	13	68	12	32	
N1+	32	66	6	32	26	68	
**Stage grouping**							*0.25*
I	4	7	2	10	2	5	
IIA	20	35	10	53	10	26	
IIB	27	47	6	33	21	55	
III	5	9	1	5	4	11	
IV	1	2	-	-	1	3	
**Differentiation**							***0.046***
Grade 1	6	11	2	11	4	11	
Grade 2	28	49	13	68	15	39	
Grade 3	22	38	3	16	19	50	
Grade 4	1	2	1	5	-	-	

Note: TNM stages and stage grouping follows AJCC 7th ed; *P* values from student's t-test for age, Fisher's exact test for dichotomous variables, or chi-square when taking all categories into account.

* A case with nuclear ATM staining was scored as "high" when there was more than 10% labelling within the tumour cell fraction and "low" when less than 10% were positive.

Supplementary References

1. Lobe CG, Koop KE, Kreppner W, Lomeli H, Gertsenstein M, Nagy A. Z/AP, a double reporter for cre-mediated recombination. *Developmental biology* **208**, 281-292 (1999).

2. Groger CJ, Grubinger M, Waldhor T, Vierlinger K, Mikulits W. Meta-analysis of gene expression signatures defining the epithelial to mesenchymal transition during cancer progression. *PloS one* **7**, e51136 (2012).

3. Cheng WY, Kandel JJ, Yamashiro DJ, Canoll P, Anastassiou D. A multi-cancer mesenchymal transition gene expression signature is associated with prolonged time to recurrence in glioblastoma. *PloS one* **7**, e34705 (2012).

4. Guzman-Ayala M, Lee KL, Mavrakis KJ, Goggolidou P, Norris DP, Episkopou V. Graded Smad2/3 activation is converted directly into levels of target gene expression in embryonic stem cells. *PloS one* **4**, e4268 (2009).
